# Use of gas chromatography mass spectrometry to elucidate metabolites predicting the phenotypes of IgA nephropathy in hyper IgA mice

**DOI:** 10.1371/journal.pone.0219403

**Published:** 2019-07-10

**Authors:** Makoto Kurano, Yutaka Yatomi

**Affiliations:** Department of Clinical Laboratory Medicine, The University of Tokyo, Tokyo, Japan; University of Bari Aldo Moro, ITALY

## Abstract

IgA nephropathy, a common chronic kidney disease, has various possible outcomes. Therefore, the identification of novel prognostic biomarkers is needed. To this purpose, we used gas chromatography mass spectrometry to search for metabolites capable of predicting the phenotypes of IgA nephropathy in hyper IgA (HIGA) mice, an established model mice for IgA nephropathy. We measured the plasma metabolite levels in 12- and 22-week-old mice, prior to the manifestation of IgA nephropathy phenotypes, and statistically investigated the associations between these metabolites and the phenotypes of IgA nephropathy, such as the urine protein levels and histological phenotypes of the kidney at 32 weeks. We observed that in plasma samples collected from 12- and 22-week-old HIGA mice, the urinary protein levels were significantly associated with 8 and 10 metabolites, the glomerular cellular component levels were significantly associated with 8 and 7 metabolites, and the mesangial substrate levels were significantly associated with 8 and 8 metabolites, respectively. Among the candidate metabolites associated with the phenotypes of IgA nephropathy, coniferyl alcohol levels were significantly higher in HIGA mice at all of the 12, 22, and 32 weeks of age. Since this study was an observational study, we could not elucidate the underlying mechanisms; however, we were able to identify new candidate metabolites, such as coniferyl alcohol, as being potentially involved in the pathogenesis of IgA nephropathy. These results might help to develop novel laboratory tests and therapeutic reagents for IgA nephropathy in the future.

## Introduction

IgA nephropathy is a common chronic kidney disease [1] characterized by predominant IgA deposition in the glomerular mesangium; the underlying mechanisms, however, have not been elucidated, although abnormal IgA and related immune-complexes have been proposed to be involved in the pathogenesis [2]. One of the clinical characteristics of IgA nephropathy is a diversity of possible outcomes; although 15%-20% of subjects with IgA nephropathy proceed to kidney failure and require hemodialysis within 10 years and 40% do so within 20 years, the stage of IgA nephropathy can progress very slowly in some subjects [3]. Therefore, the identification of prognostic biomarkers and the investigation of novel treatment targets for IgA nephropathy are important [4,5].

Hyper-IgA mice (HIGA mice) are a murine spontaneous model for IgA nephropathy [6]. The phenotypes of IgA nephropathy are reportedly observed at 25 weeks of age or thereafter, and phenotypic differences among individual mice are often observed. These characteristics of HIGA mice prompted us to search for molecules capable of predicting the phenotypes of IgA nephropathy in HIGA mice using plasma samples obtained at an age at which IgA nephropathy had not yet manifested.

Recently, advances in mass-spectrometry have enabled the simultaneous investigation of metabolites involved in many metabolic pathways, such as the glycolysis pathway, the citrate cycle, the metabolic pathways of amino acids, fatty acids, polysaccharides, and nucleic acids. Therefore, in this study, we aimed to identify metabolites capable of discriminating HIGA mice from Balb/c mice, control mice, as well as those associated with the future phenotype in HIGA mice as part of an exploratory study to identify potential prognostic biomarkers for predicting the outcome of human IgA nephropathy.

## Methods

### Mice

Female HIGA mice (n = 14) and Balb/c mice (control group, n = 7) were obtained from Japan SLC, Inc. (Shizuoka, Japan). We used this number of mice because of our previous experience of the experiments with HIGA mice [7]. The mice were anesthetized by an intraperitoneal injection of sodium pentobarbital (Somnopentyl, Kyoritsu Seiyaku Co., Tokyo, Japan) at 40 mg/kg body weight. Then, plasma samples were collected at 12 and 22 weeks of age, and plasma, urine, and kidney samples were collected at 32 weeks of age at home gage. The protocol of this experiment is described in S1 Fig. After the sampling at 32 weeks of age, the mice were euthanized by cervical dislocation without recovery from anesthesia. All the animal experiments were conducted in accordance with the guidelines for animal care and were approved by the animal committee of The University of Tokyo (protocols P11-074 and P16-044). All the mice were maintained under specific pathogen-free conditions and the mice were housed in a temperature-controlled environment with a 12-h light/dark cycle and were allowed free access to water and a standard chow diet.

### Measurement of metabolites

Each sample (25 μL) was mixed with 10 μg of 2-isopropylmalic acid (an internal standard) and 250 μL of a solvent mixture (MeOH:H_2_O:CHCl_3_ 2.5:1:1) and the solution was shaken at 1200 rpm for 30 minutes at 37°C before being centrifuged at 16,000 ×*g* for 5 minutes at 4°C. Next, 225 μL of the resultant supernatant was transferred to a clean tube, and 200 μL of distilled water was added; the solution was then centrifuged at 16,000 ×*g* for 5 minutes at 4°C. Then, 250 μL of the resultant supernatant was transferred to a clean tube and lyophilized. For oximation, 80 μL of 20-mg/mL methoxyamine hydrochloride (Sigma-Aldrich) dissolved in pyridine was mixed with a lyophilized sample, and the mixture was shaken at 1200 rpm for 90 minutes at 30°C. Next, 40 μL of N-methyl-N-trimethylsilyl-trifluoroacetamide (MSTFA) (Sigma-Aldrich) was added for derivatization, and the mixture was incubated at 1200 rpm for 30 minutes at 37°C. The mixture was then centrifuged at 16,000 ×*g* for 5 minutes, and the resultant supernatant was subjected to measurement using a gas chromatography mass spectrometry (GC-MS) system (QP2020; SHIMAZU). The levels of metabolites were measured using the Smart Metabolite Database (SHIMAZU).

### Histological analyses

For histological examination, the kidneys were resected from mice after perfusion with PBS. Formalin-fixed sections were stained with periodic acid-Schiff (PAS). To evaluate the mesangial proliferation in the glomerulus, we measured the area of the nucleus or that of the mesangial substrates using Image J and divided them by the area of the glomerulus to yield the nuclear area ratio or the mesangial area ratio. We evaluated eight glomeruli per sample.

### Measurement of urine protein levels

The urine samples were collected for 2 days. The urine total protein levels were measured using a colorimetric assay to determine protein concentration following detergent solubilization (DC protein assay, 500–0116, Bio-Rad Laboratories, Inc. Hercules, CA); the levels were then adjusted according to the urinary creatinine level, which was measured using Jaffe’s reaction (290–65901; WAKO Pure Chemical Industries). The urine protein levels were calculated as the average of the 2 days.

### Statistics

The data were statistically analyzed using SPSS (Chicago, IL) or SIMCA (MKS Umetrics). The results were expressed as the mean ± SEM. Differences between two groups were evaluated using the Student *t*-test. *P* values less than 0.05 were deemed statistically significant. The orthogonal projection to latent structures (OPLS) was statistically analyzed to explore the predicting variables for HIGA mice, urinary protein levels, and histological phenotypes (glomerular cell number and mesangial substrate area) using SIMCA (MKS Umetrics).

## Results

### Characteristics of 32-week-old HIGA mice and Balb/c mice

**Fig 1:**
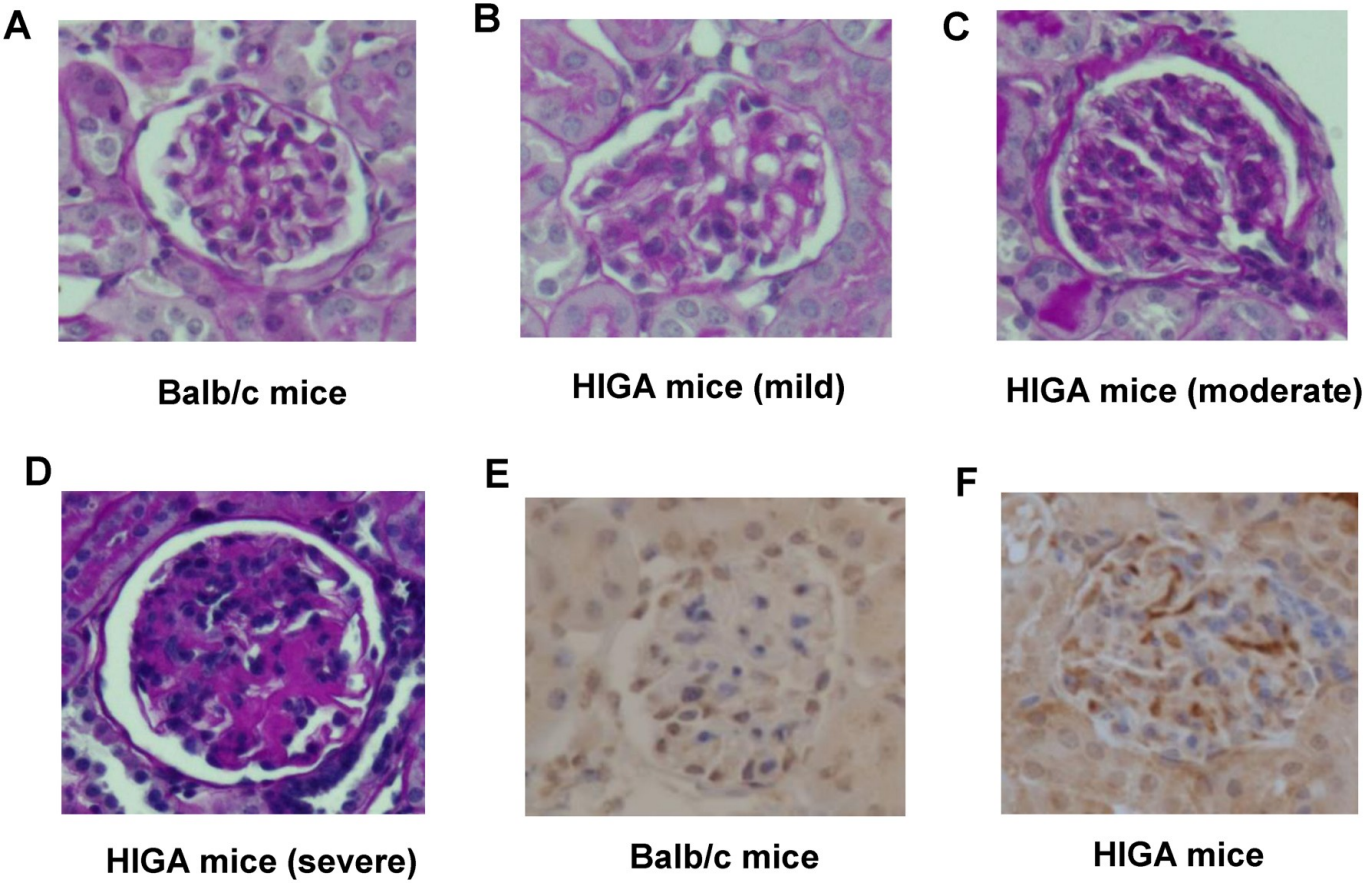
Histological characteristics of HIGA mice. The histological characteristics of 32-week-old female HIGA mice (HIGA) and Balb/c mice were analyzed using PAS staining (A–D) and IgA staining (E, F).

The characteristics of HIGA mice and Balb/c mice, which were used as a control group, are shown in Table 1. Concordant with a previous article [7], the urinary protein levels were higher in the HIGA mice. We also observed the proliferation of mesangial cells and an increase in mesangial substrates together with the deposition of IgA complex (Table 1 and Fig 1). As expected, we observed individual differences in the histological phenotypes of the HIGA mice.

**Table 1:** Characteristics of HIGA mice and Balb/c mice.

	Balb/c mice (n = 7)	HIGA mice (n = 14)
Body weight (g)	26.05 ± 0.68	48.99 ± 2.72***
Kidney weight (g)	0.42 ± 0.20	0.67 ± 0.28***
Liver weight (g)	1.24 ±0.06	2.08 ± 0.99***
Kidney/body weight (%)	1.59 ± 0.04	1.42 ± 0.08
Liver/body weight (%)	4.75 ± 0.13	4.30 ± 0.13*
Urinary protein (g/gCre)	10.20 ± 0.20	20.74 ± 0.74***
Nuclear area (%)	16.29 ± 0.28	23.45 ± 1.15***
Mesangial area (%)	40.11 ± 0.70	50.94 ± 1.33***

Data are the mean ± SEM.

*P < 0.05.

***P < 0.001.

### Metabolites in plasma samples obtained from 12-, 22-, and 32-week-old HIGA mice and Balb/c mice

In the 12-week-old and 22-week-old mice, 174 of the 568 monitored metabolites were detected, while 206 of the 568 monitored metabolites were detected in the 32-week-old mice. Among them, 58 metabolites were higher and 7 metabolites were lower in the 12-week-old HIGA mice, 62 metabolites were higher and 9 metabolites were lower in the 22-week-old HIGA mice, and 40 metabolites were higher and 62 metabolites were lower in the 32-week-old HIGA mice (S1–S3 Tables). Among the metabolites, 49 metabolites were significantly higher and 4 metabolites were significantly lower at both 12 and 22 weeks of age (S4 Table) and 16 metabolites were significantly higher and 2 metabolites were significantly lower at all of the 12, 22, and 32 weeks of age (Table 2).

**Table 2:** Metabolites significantly modulated in HIGA mice in all of the 12, 22, and 32 week old.

A. Metabolites significantly higher in HIGA mice in all of the 12, 22, and 32 week old.						
	12 week old		22 week old		32 week old	
**Metabolites**	**HIGA/Balb/c**	**p-value**	**HIGA/Balb/c**	**p-value**	**HIGA/Balb/c**	**p-value**
Glucuronic acid-5TMS(2)	2.89541	<0.00001	2.49209	<0.00001	2.06108	<0.00001
Inositol phosphate-7TMS	1.53509	0.00010	1.75006	0.00099	1.85005	0.00009
Myristic acid-TMS	1.82336	0.00015	1.66276	0.00010	1.53618	0.00022
Xylitol-5TMS	1.45141	0.00017	1.45456	0.00008	1.82945	0.00010
Indol-3-acetic acid-TMS	1.25844	0.00215	1.21621	0.00509	1.18954	0.03450
Inosine-4TMS	20.18891	0.00415	18.56310	0.00001	2.17511	0.00683
Galactitol-6TMS	1.83617	0.00274	2.07812	0.00011	1.44513	0.03552
Coniferyl alcohol-2TMS	1.34618	0.00320	1.23151	0.02318	1.19172	0.03570
Galactosamine-5TMS(1)	1.66865	0.00442	1.24245	0.00229	1.46790	0.00190
Glucosamine-5TMS(1)	1.21603	0.00979	1.50628	0.00246	1.25321	0.02218
Tyramine-3TMS	1.20258	0.01817	1.28135	0.00231	1.24799	0.03695
5-Dehydroquinic acid-5TMS	1.28379	0.02403	1.28189	0.02267	1.44342	0.00295
1,5-Anhydro-glucitol-4TMS	1.23604	0.03318	1.25176	0.01222	1.23455	0.01073
Galactose-5TMS(2)	1.21511	0.03987	1.26687	0.00203	1.25862	0.00074
Mannose-5TMS(2)	1.21511	0.03987	1.26687	0.00203	1.25862	0.00074
Allose-5TMS	1.21511	0.03987	1.26687	0.00203	1.25862	0.00074
B. Metabolites significantly lower in HIGA mice in all of the 12, 22, and 32 week old.						
Metabolites	HIGA/Balb/c	p-value	HIGA/Balb/c	p-value	HIGA/Balb/c	p-value
Tyrosine-3TMS	0.49751	<0.00001	0.54995	0.02270	0.81751	0.03122
Hypotaurine-3TMS	0.74505	0.03020	0.79055	0.04874	0.35015	0.00033

**Fig 2:**
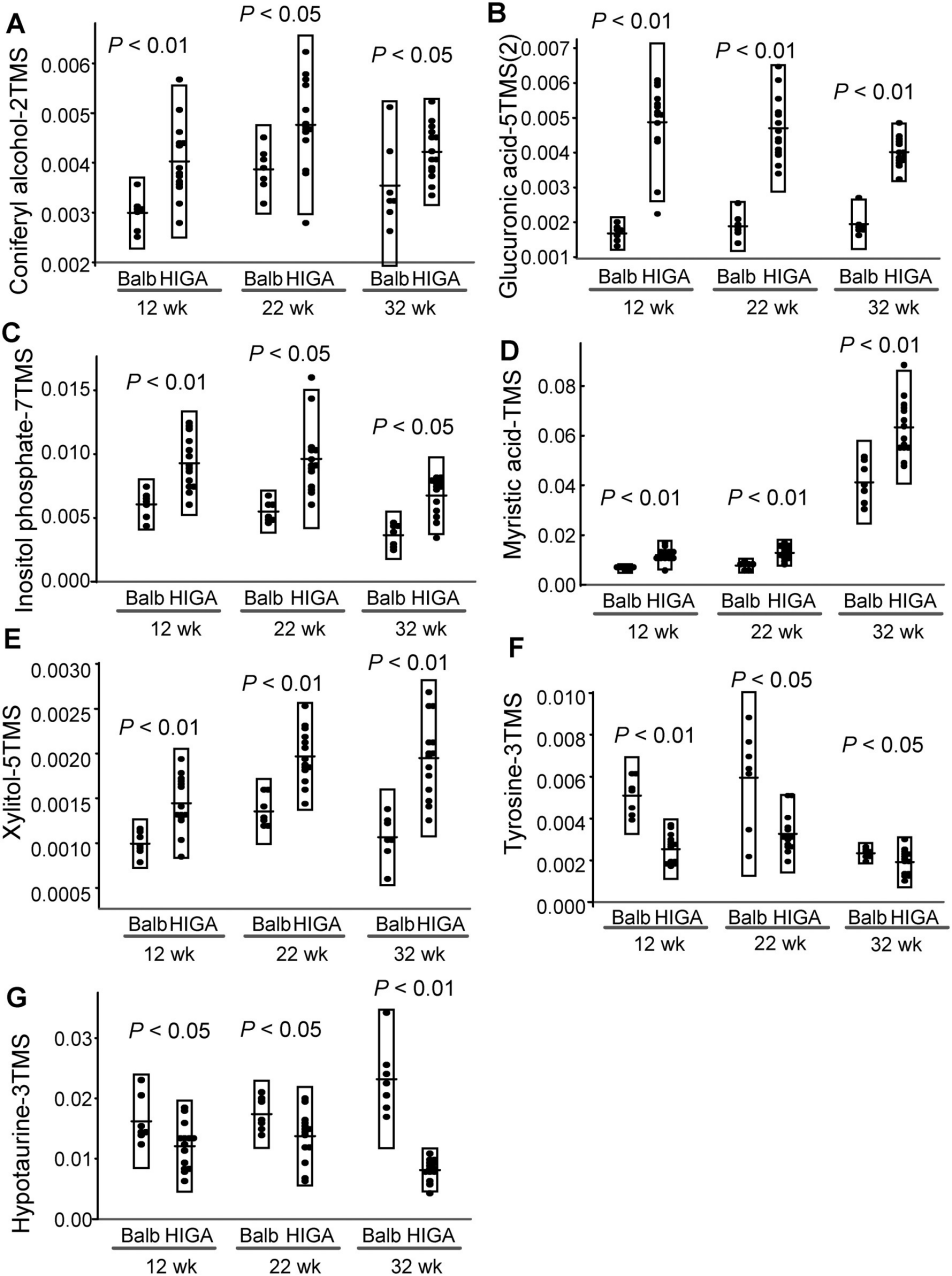
Time courses for the levels of representative metabolites in HIGA mice. The time courses for the levels of representative metabolites (coniferyl alcohol, glucuronic acid, inositol phosphate, myristic acid, xylitol, tyrosine, and hypotaurine), which were modulated similarly at all of the 12, 22, and 32 weeks of age, are shown. The data are shown as the ratio to an internal standard (2-isopropylmalic acid at 25 μg/μL).

OPLS analyses revealed that these metabolites were capable of discriminating HIGA mice from Balb/c mice (S2–S4 Figs). The metabolites with high variable importance in the projection for HIGA mice are shown in Table 3. As shown in Table 3A, glucuronic acid was selected as the most positive predictive variable, followed by uracil, monosaccharides (such as mannose, allose, and psicose), and myristic acid, while several amino acids (such as tyrosine, tryptophan, aspartic acid, and serine), coniferyl aldehyde, and 2-hydroxybutyric acid were selected as significant negative predictive variables at the age of 12 weeks. At 22 weeks, at which time the phenotypes of IgA nephropathy had not yet become apparent, the selected metabolites were not so different from those in 12-week-old mice; glucuronic acid was still selected as the most positive predictive variable, followed by xylitol, xanthine, inosine, threonic acid, and myristic acid. Regarding the negative predictive variables, several amino acids (such as tyrosine, aspartic acid, glycine, and O-acetyl-serine) and coniferyl aldehyde were selected (Table 3B). In the 32-week-old mice, at which time the phenotypes of IgA nephropathy had become apparent, glucuronic acid was still selected as the most positive predictive variable, followed by alanine, leucine, inositol phosphate, xylitol, and myristic acid, while some metabolites of glucose metabolism (such as phosphoenolpyruvic acid, glycerol 3-phosphate, and oxalic acid), hypotaurine, O-phosphoethanolamine, and eicosapentaenoic were selected as negative predictive variables. The time courses of the levels of representative metabolites (glucuronic acid, inositol phosphate, myristic acid, xylitol, tyrosine, and hypotaurine), which were modulated similarly at all of the 12, 22, and 32 weeks of age, are shown in Fig 2.

**Table 3:** OPLS analysis of metabolites with variable importance in projection for HIGA mice.

A. 12 weeks			
Positive predicting variable		Negative predicting variable	
Metabolites	VIP	Metabolites	VIP
Glucuronic acid-5TMS(2)	0.1504 ± 0.0226	Tyrosine-3TMS	-0.1496 ± 0.0611
Uracil-2TMS	0.1498 ± 0.0451	Coniferyl aldehyde-meto-TMS	-0.1204 ± 0.0816
Mannose-meto-5TMS(1)	0.1393 ± 0.0300	Tryptophan-3TMS	-0.0781 ± 0.0668
Allose-meto-5TMS(1)	0.1389 ± 0.0310	Aspartic acid-3TMS	-0.0769 ± 0.0709
Psicose-5TMS(1)	0.1324 ± 0.0482	2-Hydroxybutyric acid-2TMS	-0.0682 ± 0.0573
Myristic acid-TMS	0.1280 ± 0.0334	Serine-3TMS	-0.0544 ± 0.0511
B. 22 weeks			
Positive predicting variable		Negative predicting variable	
Metabolites	VIP	Metabolites	VIP
Glucuronic acid-5TMS(2)	0.1488 ± 0.0443	Tyrosine-3TMS	-0.1161 ± 0.1004
Xylitol-5TMS	0.1350 ± 0.0407	Aspartic acid-3TMS	-0.1057 ± 0.0480
Xanthine-3TMS	0.1350 ± 0.0377	Coniferyl aldehyde-meto-TMS(1)	-0.1032 ± 0.0810
Inosine-4TMS	0.1337 ± 0.0575	Threonine-3TMS	-0.0940 ± 0.0882
Threonic acid-4TMS	0.1326 ± 0.0420	O-Acetylserine-2TMS	-0.0877 ± 0.0874
Myristic acid-TMS	0.1303 ± 0.0521	Glycine-3TMS	-0.0868 ± 0.0786
C. 32 weeks			
Positive predicting variable		Negative predicting variable	
Metabolites	VIP	Metabolites	VIP
Glucuronic acid-5TMS(2)	0.1236 ± 0.0564	Phosphoenolpyruvic acid-3TMS	-0.1229 ± 0.0519
Alanine-2TMS	0.1015 ± 0.0440	Hypotaurine-3TMS	-0.1206 ± 0.0483
Leucine-2TMS	0.0996 ± 0.0420	Glycerol 3-phosphate-4TMS	-0.1185 ± 0.0298
Inositol phosphate-7TMS	0.0994 ± 0.0335	O-Phosphoethanolamine-4TMS	-0.1165 ± 0.0346
Xylitol-5TMS	0.0982 ± 0.0467	Eicosapentaenoic acid-TMS	-0.1150 ± 0.0355
Myristic acid-TMS	0.0940 ± 0.0299	Oxalic acid-2TMS	-0.1137 ± 0.0318

Data are the mean ± SEM. VIP means variable importance in projection, which was calculated in OPLS analyses using SIMCA.

### Several metabolites in plasma samples obtained from 12-, 22-, or 32-week-old HIGA mice were identified as possible predictors of urine protein levels in HIGA mice

Next, we investigated possible substances capable of predicting the urine protein levels at 32 weeks using plasma samples obtained from 12-, 22-, and 32-week old HIGA mice. As shown in S5 Fig and Table 4, OPLS analyses identified several significant predictive substances. In 12-week-old mice, coniferyl alcohol was selected as the only positive significant predictive substance, while six substances were selected as negative significant predictive variables: 3 amino acids (glycine, proline, and methionine), and 3 monosaccharides (sorbose, psicose, and thiodiglycolic acid) (Table 4A). In 22-week-old mice, fucose and galacturonic acid were selected as positive predictive substances, while gluconic acid, glyceraldehyde, glyoxylic acid, lysine, isoleucine, allo-isoleucine, 4-aminobutyric acid, and thiodiglycolic acid were selected as negative significant predictive substances (Table 4B). In 32-week-old mice, aconitic acid and citric acid, which are metabolites in the TCA cycle, were selected as positive significant predictors as well as myristic acid, while serine, propionylglycine, 4-hydroxybutyric acid, and rhamnose were selected as negative predictors (Table 4C).

**Table 4:** OPLS analysis of metabolites with variable importance in projection for urinary protein levels.

A. 12 weeks			
Positive predicting variable		Negative predicting variable	
Metabolites	VIP	Metabolites	VIP
Coniferyl alcohol-2TMS	0.0864 ± 0.0678	Glycine-3TMS	-0.1486 ± 0.0766
		Proline-2TMS	-0.1460 ± 0.0796
		Methionine-2TMS	-0.1445 ± 0.0439
		Sorbose-meto-5TMS(1)	-0.1410 ± 0.0542
		Thiodiglycolic acid-2TMS	-0.1392 ± 0.0551
		Psicose-meto-5TMS(2)	-0.1387 ± 0.0411
B. 22 weeks			
Positive predicting variable		Negative predicting variable	
Metabolites	VIP	Metabolites	VIP
Fucose-4TMS(1)	0.1386 ± 0.0951	Gluconic acid-6TMS	-0.1638 ± 0.0937
Galacturonic acid-5TMS(1)	0.1029 ± 0.0949	Glyceraldehyde-meto-2TMS(2)	-0.1426 ± 0.1055
		Lysine-4TMS	-0.1389 ± 0.0643
		Glyoxylic acid-meto-TMS	-0.1387 ± 0.1020
		Isoleucine-2TMS	-0.1324 ± 0.1177
		allo-Isoleucine-2TMS	-0.1324 ± 0.1177
		4-Aminobutyric acid-2TMS	-0.1324 ± 0.1177
		Thiodiglycolic acid-2TMS	-0.1283 ± 0.1037
C. 32 weeks			
Positive predicting variable		Negative predicting variable	
Metabolites	VIP	Metabolites	VIP
Aconitic acid-3TMS	0.1524 ± 0.0705	Serine-3TMS	-0.1338 ± 0.1136
Myristic acid-TMS	0.1188 ± 0.0505	Propionylglycine-TMS	-0.1330 ± 0.1247
Citric acid-d4-4TMS	0.1073 ± 0.1070	4-Hydroxybutyric acid-2TMS	-0.1082 ± 0.1046
		Rhamnose-4TMS(1)	-0.0966 ± 0.0859

Data are the mean ± SEM. VIP means variable importance in projection, which was calculated in OPLS analyses using SIMCA.

### Several metabolites in plasma samples obtained from 12-, 22-, or 32-week-old HIGA mice were identified as possible predictors of histological phenotypes in HIGA mice

Finally, we searched for metabolites capable of predicting two histological phenotypes of IgA nephropathy, glomerular cell number (Table 5) and mesangial substrate deposition (Table 6), in 32-week-old HIGA mice.

**Table 5:** OPLS analysis of metabolites with variable importance in projection for glomerular cellular component levels.

A. 12 weeks			
Positive predicting variable		Negative predicting variable	
Metabolites	VIP	Metabolites	VIP
Glycerol-3TMS	0.1047 ± 0.1016	Propionylglycine-TMS	-0.1934 ± 0.1188
Linoleic acid-TMS	0.0832 ± 0.0825	Fructose-meto-5TMS(1)	-0.1815 ± 0.0557
		Sorbose-meto-5TMS(1)	-0.1800 ± 0.0665
		Psicose-meto-5TMS(2)	-0.1663 ± 0.0464
		Pantothenic acid-3TMS	-0.1559 ± 0.1047
		Proline-2TMS	-0.1452 ± 0.1035
B. 22 weeks			
Positive predicting variable		Negative predicting variable	
Metabolites	VIP	Metabolites	VIP
Taurine-3TMS	0.1926 ± 0.0978	Glyoxylic acid-meto-TMS	-0.0852 ± 0.0604
Taurine-13C2-3TMS	0.1916 ± 0.0998		
Mannose 6-phosphate-meto-6TMS(1)	0.1806 ± 0.1347		
5-Methoxytryptamine-2TMS	0.1765 ± 0.0832		
Dihydroorotic acid-3TMS	0.1732 ± 0.1021		
Spermidine-5TMS	0.1730 ± 0.1203		
C. 32 weeks			
Positive predicting variable		Negative predicting variable	
Metabolites	VIP	Metabolites	VIP
Glycine-2TMS	0.1230 ± 0.1196	Cystamine-nTMS	-0.1850 ± 0.1667
Dimethylglycine-TMS	0.1100 ± 0.0883	5-Oxoproline-2TMS	-0.1787 ± 0.1074
Boric acid-3TMS	0.1066 ± 0.0842	Tryptophan-3TMS	-0.1754 ± 0.1232
Xylitol-5TMS	0.0725 ± 0.0449	Hypoxanthine-2TMS	-0.1650 ± 0.1068
		Xanthine-3TMS	-0.1329 ± 0.0911
		Rhamnose-4TMS(1)	-0.1312 ± 0.1015

Data are the mean ± SEM. VIP means variable importance in projection, which was calculated in OPLS analyses using SIMCA.

**Table 6:** OPLS analysis of metabolites with variable importance in projection for mesangial substrate levels.

A. 12 weeks			
Positive predicting variable		Negative predicting variable	
Metabolites	VIP	Metabolites	VIP
Coniferyl alcohol-2TMS	0.0732 ± 0.0513	Valine-2TMS	-0.1603 ± 0.0520
3,6-Epoxydodecanedioic acid-2TMS	0.0627 ± 0.0473	Leucine-2TMS	-0.1575 ± 0.0317
		Methylmalonic acid-2TMS	-0.1571 ± 0.0777
		Phenylalanine-2TMS	-0.1568 ± 0.0586
		Glyceraldehyde-meto-2TMS(2)	-0.1560 ± 0.0576
		Urea-2TMS	-0.1457 ± 0.0701
B. 22 weeks			
Positive predicting variable		Negative predicting variable	
Metabolites	VIP	Metabolites	VIP
3-Hydroxyisobutyric acid-2TMS	0.1410 ± 0.1369	Stearic acid-TMS	-0.1929 ± 0.0680
Hydroxylamine-3TMS	0.1092 ± 0.0906	Margaric acid-TMS	-0.1840 ± 0.1002
		Glucono-1,4-lactone-4TMS	-0.1531 ± 0.1254
		Palmitic acid-TMS	-0.1322 ± 0.0664
		Glyceraldehyde-meto-2TMS(2)	-0.1282 ± 0.0905
		Ribitol-5TMS	-0.1255 ± 0.1127
C. 32 weeks			
Positive predicting variable		Negative predicting variable	
Metabolites	VIP	Metabolites	VIP
Xylitol-5TMS	0.1185 ± 0.0885	Tryptophan-3TMS	-0.1663 ± 0.0919
Allose-meto-5TMS(1)	0.0923 ± 0.0597	5-Oxoproline-2TMS	-0.1600 ± 0.1392
Mannose-meto-5TMS(1)	0.0908 ± 0.0585	Leucine-2TMS	-0.1346 ± 0.1021
		Isoleucine-2TMS	-0.1214 ± 0.0955
		Ornithine-4TMS	-0.1208 ± 0.1054
		Ribose-meto-4TMS	-0.1187 ± 0.0834

Data are the mean ± SEM. VIP means variable importance in projection, which was calculated in OPLS analyses using SIMCA.

For the glomerular cell number, glycerol and linoleic acid were selected as positive predictive substances in 12-week-old mice, while propionylglycine, pantothenic acid, proline, and some monosaccharides (such as fructose, sorbose, and psicose) were selected as negative predictive substances (Table 5A and S6A Fig). In 22-week-old mice, taurine, mannose 6-phosphate, 5-methoxytryptamine, dihydroorotic acid, and spermidine were selected as positive predictive substrates, while only glyoxylic acid was selected as a negative predictive substance (Table 5B and S6B Fig). In 32-week-old mice, glycine, dimethylglycine, boric acid, and xylitol were selected as positive predictive substances, while cystamine, 5-oxoproline, tryptophan, hypoxanthine, xanthine, and rhamnose were selected as negative predictive substances (Table 5C and S6C Fig).

Regarding the mesangial substrate levels, coniferyl alcohol and epoxydodecanedioic acid were selected as positive predictive substances in 12-week-old mice, while several amino acids (valine, leucine, phenylalanine), methylmalonic acid, glyceraldehyde, and urea were selected as negative predictive substances (Table 6A and S7A Fig). In 22-week-old mice, 3-hydroxyisobutyric acid and hydroxylamine were selected as positive predictive substances for the expansion of mesangial substrates, while several fatty acids (stearic acid, margaric acid, palmitic acid), glucono-1,4-lactone, glyceraldehyde, and ribitol were selected as significant negative predictive substances (Table 6B, S7B Fig). In 32-week-old mice, several monosaccharides, such as xylitol, allose, and mannose, were selected as positive predictive substances, while several amino acids (such as tryptophan, leucine, isoleucine, and 5-oxoproline), ornithine, and ribose were selected as negative predictive substances (Table 6C, S7C Fig).

## Discussion

The search for predictive biomarkers for the phenotypes of IgA nephropathy is an important task, considering the various possible outcomes of IgA nephropathy. Metabolic biomarkers for IgA nephropathy have been previously investigated in human subjects [8,9], although few studies have identified any biomarkers capable of predicting the outcome of IgA nephropathy. One of the difficulties in the search for predictive biomarkers is that IgA nephropathy, in general, proceeds slowly in human subjects. Therefore, in this study, we aimed to identify metabolites capable of predicting the phenotypes of IgA nephropathy in HIGA mice, in which the disease becomes apparent within 7 months, as an exploratory study to identify novel biomarkers or therapeutic targets for human IgA nephropathy. We used a GC-MS method to measure many plasma metabolites simultaneously and found that several metabolites in 12- and 22-week-old mice, in which the phenotypes of IgA nephropathy have not yet become obvious, could predict the urinary protein levels and renal histological phenotypes in 32-week-old mice, in which the disease phenotype has been established. Among the predicting metabolites for the phenotypes of IgA nephropathy, which we identified in the present study, we discuss on the metabolites, which were higher or lower at all of the 12, 22, and 32 weeks of age since these metabolites might be more reliable than those modulated in one or two points. Considering the possibility that several metabolite, which were modulated in both 12-week-old and 22-week-old HIGA mice but not in 32-week-old HIGA mice, might be compensatory modulated in 32 weeks old, when the phenotypes of HIGA mice had been established, we also discussed on the metabolites, which were higher or lower only in both 12-week-old and 22-week-old HIGA mice.

Regarding the urinary protein level in 32-week-old HIGA mice, we observed one positive predicting metabolite and six negative ones in 12-week-old HIGA mice and two positive predicting metabolites and eight negative ones in 22-week-old HIGA mice. Of these possible predicting metabolites, only coniferyl alcohol was modulated similarly at all of the 12, 22, and 32 weeks of age (Fig 2A). Coniferyl alcohol predicted the urinary protein level at 32 weeks in the 12-week-old HIGA mice. An association between coniferyl alcohol and IgA nephropathy or even kidney disease has not been previously demonstrated. As for the biological properties of coniferyl alcohol, although only limited reports exist, coniferyl alcohol reportedly inhibits apoptosis and inflammation in macrophages [10]. Among the metabolites which were similarly higher or lower only in both 12-week-old and 22-week-old HIGA mice, we observed that the fucose levels in 22-week-old HIGA mice predicted the urinary protein level at 32 weeks. Although no reports have demonstrated the association between plasma fucose levels and IgA nephropathy, the aberrant IgA1 was reportedly galactosylated with the fucose residue in the serum obtained from IgA nephropathy patients [11].

Regarding the histological phenotypes of IgA nephropathy, we also searched for metabolites capable of predicting the nuclear ratio, which represents the number of cells, including mesangial cells, in the glomerulus, and the mesangial area ratio, which represents the expansion of mesangial substrates. For the glomerular cellular component levels, we observed two positively predictive metabolites and six negative ones in 12-week-old mice, while six positive metabolites and one negative metabolite were observed in 22-week-old mice (Table 5A and 5B). Among them, no metabolites were significantly modulated at all of the 12, 22, and 32 weeks of age, while the pantothenic acid, taurine, and dihydroorotic acid levels were higher in the HIGA mice in both 12 week and 22 week old. In the 22-week-old mice, taurine was selected as the most predictive metabolite. Although taurine has not been reported to be associated with IgA nephropathy, several reports have demonstrated the possible protective properties of taurine in patients with kidney diseases through the attenuation of endoreticular stress [12,13]. As for pantothenic acid and dihydroorotic acid, no previous reports have demonstrated their involvement in kidney diseases. Regarding the expansion of the mesangial area, we observed two positive predicting metabolites and six negative ones in 12-week-old HIGA mice and two positive predicting metabolites and six negative ones in 22-week-old HIGA mice. Among the possible predicting metabolites for the expansion of the mesangial area, only coniferyl alcohol was higher at all of the 12, 22, and 32 weeks of age. Considering the results in the predicting metabolites for urinary protein levels, coniferyl alcohol might be a promising candidate to investigate its association with IgA nephropathy in the future.

In addition to the metabolites which were constantly higher or lower in HIGA mice, we observed several metabolites which were higher or lower only in 12-week-old HIGA mice or in 22-week-old HIGA mice. Since this is an observational study, we could not elucidate the significance of these metabolites. One possible reason is that the results might be partly affected by the precision in the analyses with mass spectrometry, which is in general lower than laboratory tests with automatic analyzer. Especially, in this study, we simultaneously measured fine substances using a semi-quantitative analysis without standard curves, since the aim of the present study was to identify candidate metabolites for screening. Another possible reason is that the metabolites might possess several physiological properties in the early stage and then compensatory modulated in 22 week old or that they might play several roles in the pathogenesis of IgA nephropathy only from about 22 week old. Actually, among the metabolites which were significantly modulated only in 12 week or 22 week old, some amino acids, monosaccharides, fatty acids, glycerol, mannose 6-phosphate, and spermidine were included, which have been reported to be involved in the kidney diseases. Further studies investigating the effects of the metabolites on IgA nephropathy are necessary to address this issue.

The limitations of the present study are that we could not elucidate the underlying mechanisms or validate the results in human subjects. Interpreting the results was especially difficult, as we could not determine which of the selected predictive metabolites might be involved in the pathogenesis of IgA nephropathy or whether their plasma levels might be modulated by several unidentified mechanisms involved in IgA nephropathy. Moreover, although we used HIGA mice as an animal model of mesangial proliferative glomerulonephritis, their pathogenesis and/or the modulation of metabolites might be different from human IgA nephropathy. Regardless of these limitations, the GC-MS analyses revealed several metabolites whose possible involvements in the pathogenesis of IgA nephropathy have not been previously proposed. Since several metabolites previously reported to be associated with IgA nephropathy and/or kidney disease, such as monosaccharides, amino acids, fatty acids, and taurine, were successfully selected in the present study, the novel candidate metabolites revealed in the present study might also be involved in the pathogenesis of IgA nephropathy. Further studies are required to investigate whether these metabolites might be modulated in human subjects and how they might be involved in the pathogenesis of IgA nephropathy.

In summary, we identified novel candidate metabolites capable of predicting the progression of phenotypes of IgA nephropathy, such as the urine protein level and pathological renal phenotypes, in HIGA mice. Among them, coniferyl alcohol was the most promising candidate metabolite. These results might aid the development of novel laboratory tests and therapeutic reagents for IgA nephropathy in the future.

## Supporting information

S1 Fig Protocol for the experiments with HIGA mice. (TIF)

S2 Fig OPLS analysis for metabolites with variable importance in projection in 12-week-old HIGA mice. An OPLS analysis was performed to investigate metabolites with variable importance in projection in 12-week-old HIGA mice. (A) Loading scatter plot. (B) Loading column plot.
(TIF)

S3 Fig OPLS analysis for metabolites with variable importance in projection in 22-week-old HIGA mice. An OPLS analysis was performed to investigate metabolites with variable importance in projection in 22-week-old HIGA mice. (A) Loading scatter plot. (B) Loading column plot.
(TIF)

S4 Fig OPLS analysis for metabolites with variable importance in projection in 32-week-old HIGA mice. An OPLS analysis was performed to investigate metabolites with variable importance in projection in 32-week-old HIGA mice. (A) Loading scatter plot. (B) Loading column plot.
(TIF)

S5 Fig OPLS analysis for metabolites with variable importance in projection for urinary protein levels. An OPLS analysis was performed to investigate metabolites with variable importance in projection for urinary protein levels at 32 weeks in 12-week-old mice (A), 22-week-old mice (B), and 32-week-old mice (C). Loading column plots are shown.
(TIF)

S6 Fig OPLS analysis for metabolites with variable importance in projection for glomerular cellular component levels. An OPLS analysis was performed to investigate metabolites with variable importance in projection for glomerular cellular component levels at 32 weeks in 12-week-old mice (A), 22-week-old mice (B), and 32-week-old mice (C). Loading column plots are shown.
(TIF)

S7 Fig OPLS analysis for metabolites with variable importance in projection for mesangial substrate levels. An OPLS analysis was performed to investigate metabolites with variable importance in projection for mesangial substrate levels at 32 weeks in 12-week-old mice (A), 22-week-old mice (B), and 32-week-old mice (C). Loading column plots are shown.
(TIF)

S1 Table Metabolites that differed significantly between HIGA mice and Balb/c mice at 12 weeks. Metabolites that were significantly higher (A) or lower (B) in 12-week-old HIGA mice, compared with control Balb/c mice, are shown. The levels of metabolites are shown as the ratio to an internal standard (2-isopropylmalic acid at 25 μg/μL).
(PDF)

S2 Table Metabolites that differed significantly between HIGA mice and Balb/c mice at 22 weeks. Metabolites that were significantly higher (A) or lower (B) in 22-week-old HIGA mice, compared with control Balb/c mice, are shown. The levels of metabolites are shown as the ratio to an internal standard (2-isopropylmalic acid at 25 μg/μL).
(PDF)

S3 Table Metabolites that differed significantly between HIGA mice and Balb/c mice at 32 weeks. Metabolites that were significantly higher (A) or lower (B) in 32-week-old HIGA mice, compared with control Balb/c mice, are shown. The levels of metabolites are shown as the ratio to an internal standard (2-isopropylmalic acid at 25 μg/μL).
(PDF)

S4 Table Metabolites significantly modulated in HIGA mice in both 12 week and 22 week old. Metabolites that were significantly higher (A) or lower (B) in both 12- and 22-week-old HIGA mice, compared with control Balb/c mice, are shown.
(PDF)

S1 Dataset Dataset-PONE-D-19-07913.xlsx. This data set shows the plasma concentrations of the metabolites in the HIGA mice and the Balb/c mice.
(XLSX)
