# Supplementary figures and images for: Use of gas chromatography mass spectrometry to elucidate metabolites predicting the phenotypes of IgA nephropathy in hyper IgA mice
Source: PLoS One. 2019 Jul 10;14(7):e0219403. doi: 10.1371/journal.pone.0219403 (PMC6619804; doi:10.1371/journal.pone.0219403)

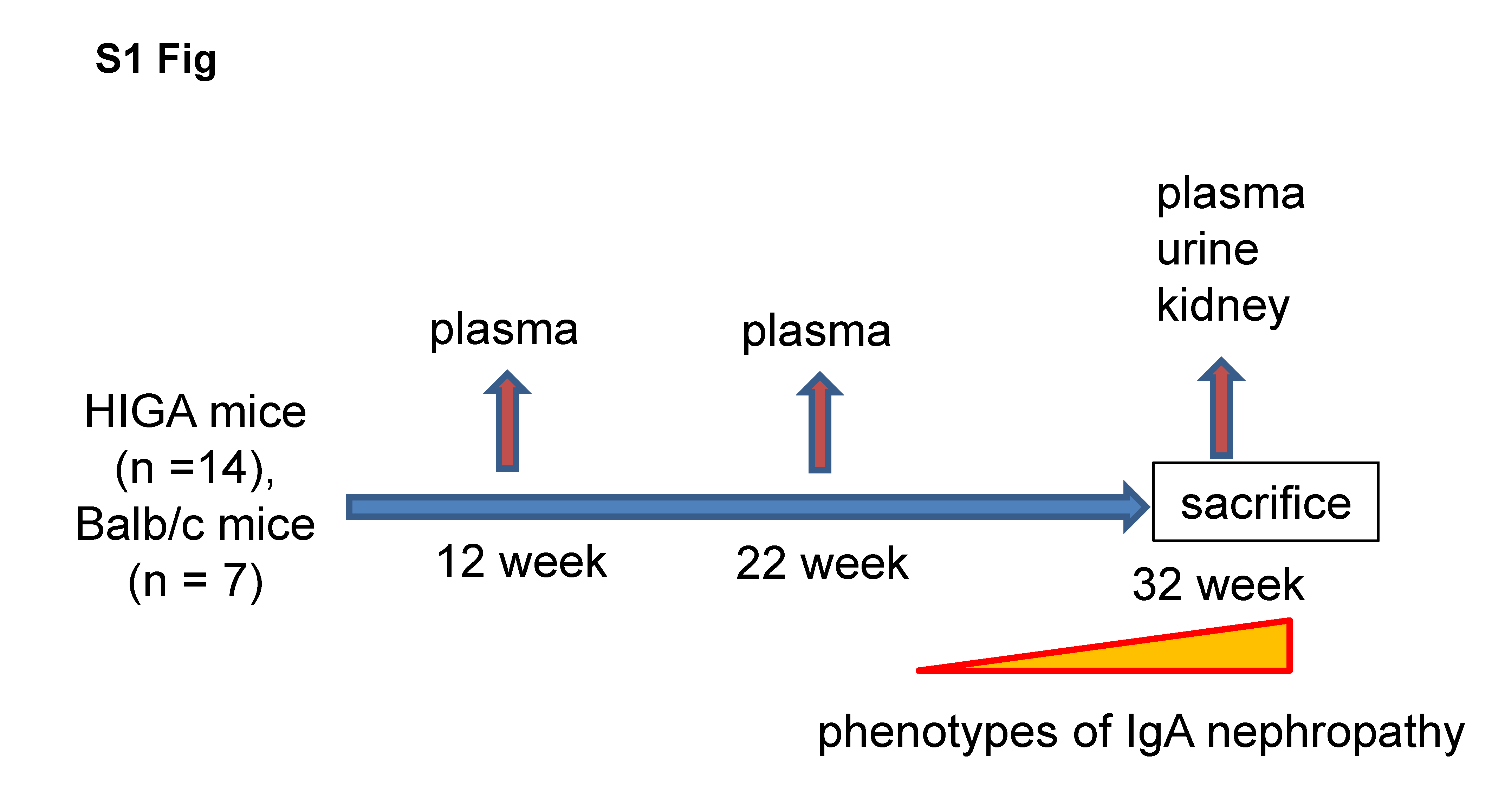

Supplement: S1 Fig — (TIF) [file pone.0219403.s001.tif]

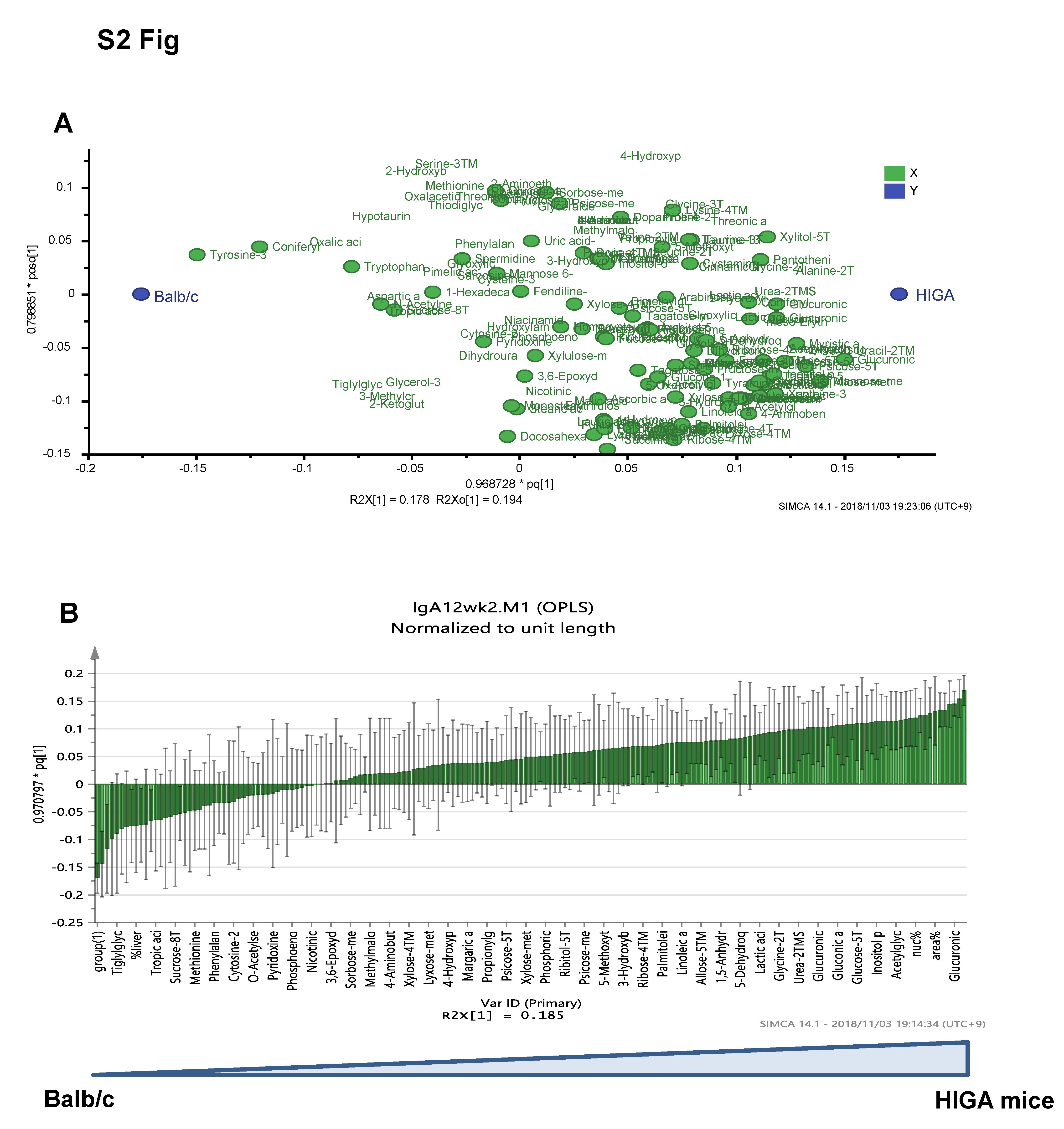

Supplement: S2 Fig — An OPLS analysis was performed to investigate metabolites with variable importance in projection in 12-week-old HIGA mice. (A) Loading scatter plot. (B) Loading column plot. (TIF) [file pone.0219403.s002.tif]

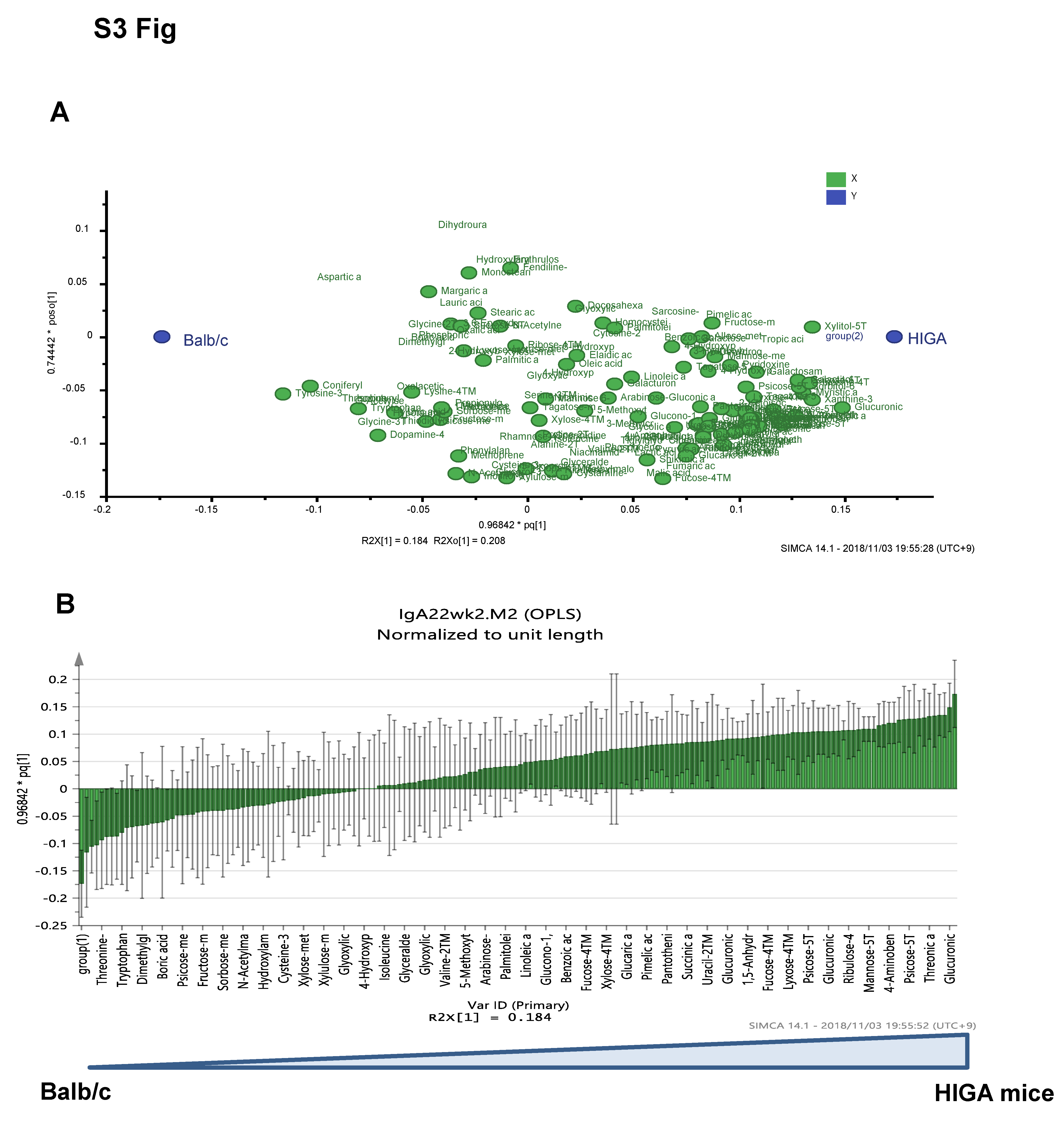

Supplement: S3 Fig — An OPLS analysis was performed to investigate metabolites with variable importance in projection in 22-week-old HIGA mice. (A) Loading scatter plot. (B) Loading column plot. (TIF) [file pone.0219403.s003.tif]

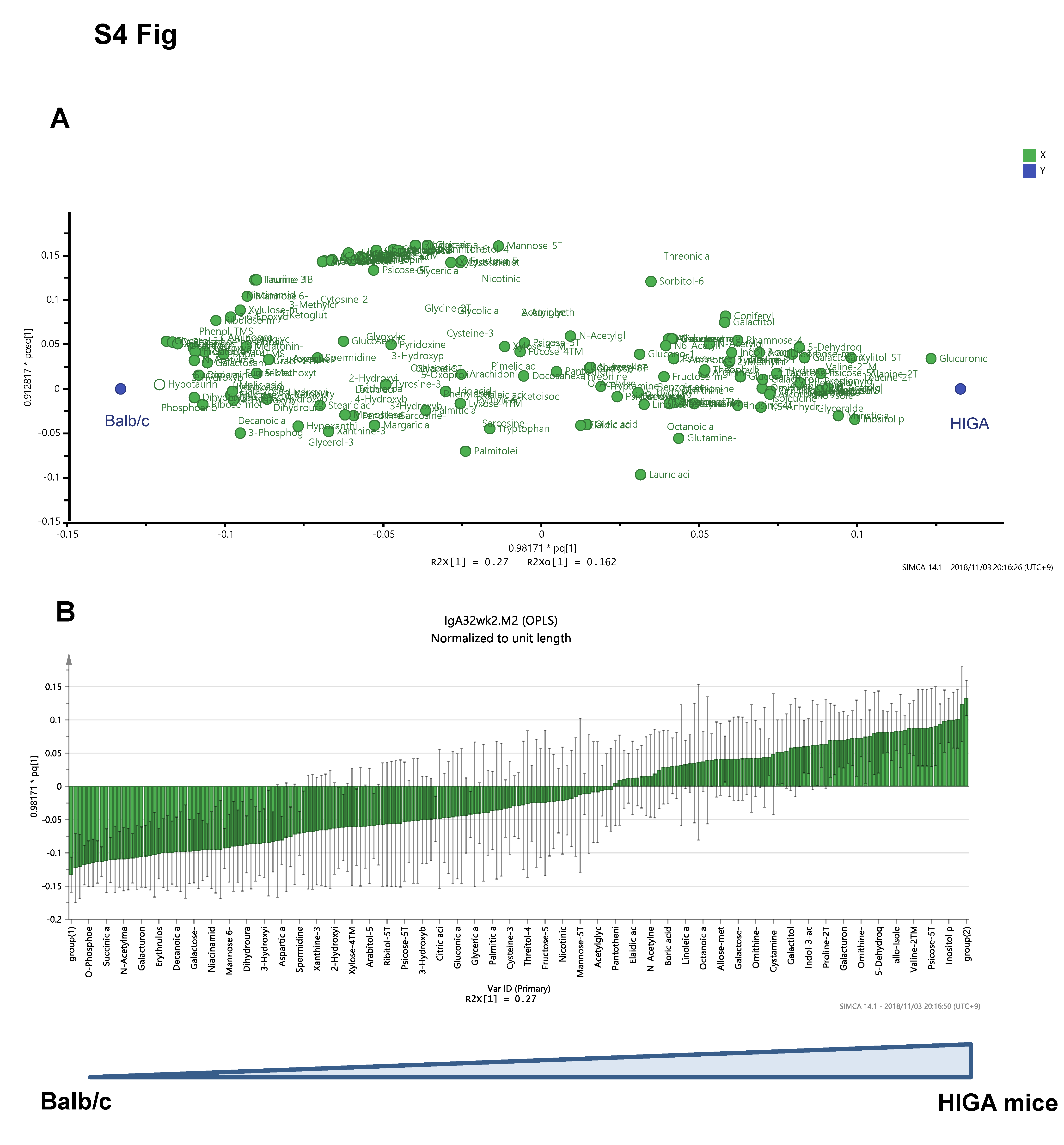

Supplement: S4 Fig — An OPLS analysis was performed to investigate metabolites with variable importance in projection in 32-week-old HIGA mice. (A) Loading scatter plot. (B) Loading column plot. (TIF) [file pone.0219403.s004.tif]

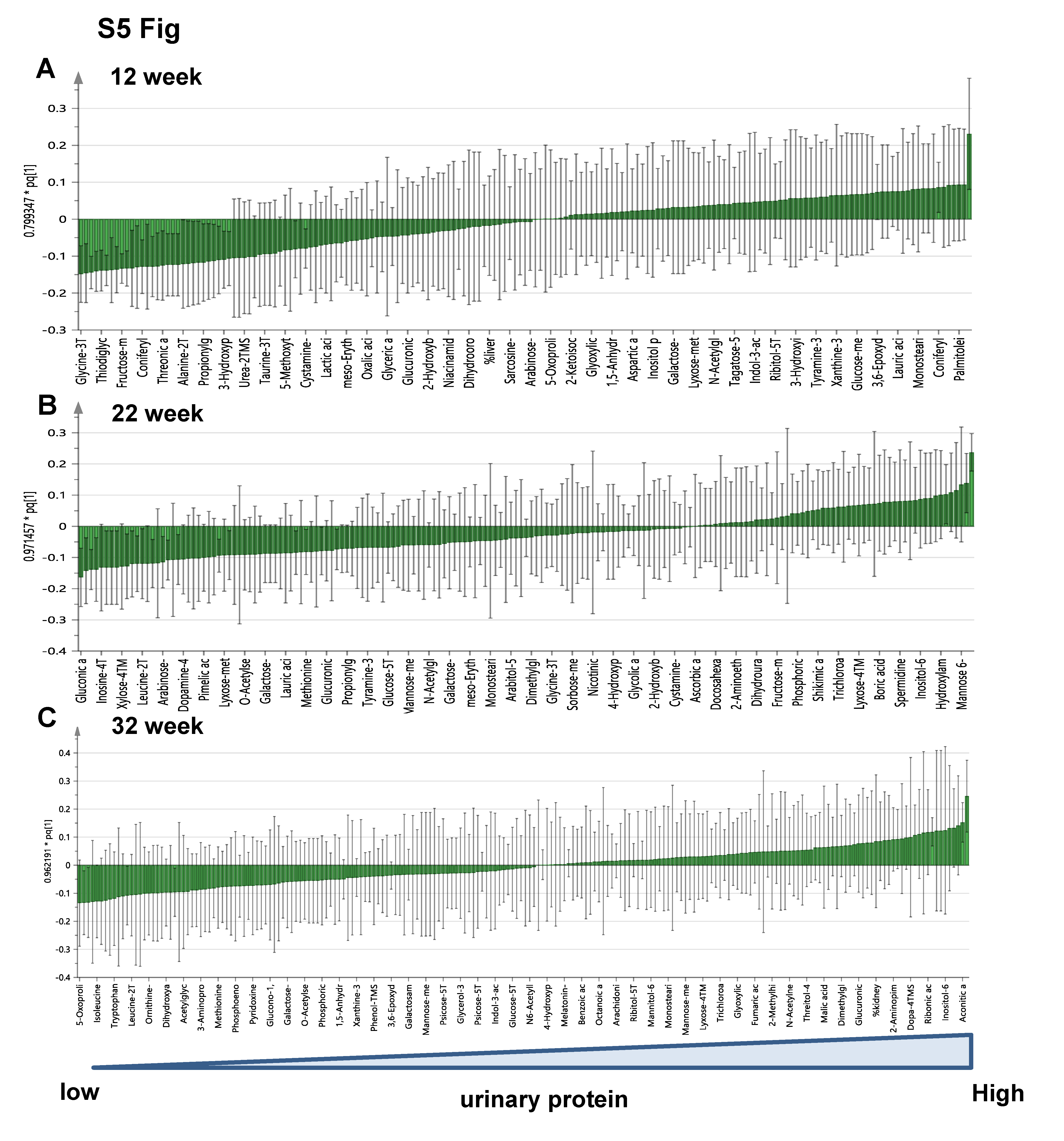

Supplement: S5 Fig — An OPLS analysis was performed to investigate metabolites with variable importance in projection for urinary protein levels at 32 weeks in 12-week-old mice (A), 22-week-old mice (B), and 32-week-old mice (C). Loading column plots are shown. (TIF) [file pone.0219403.s005.tif]

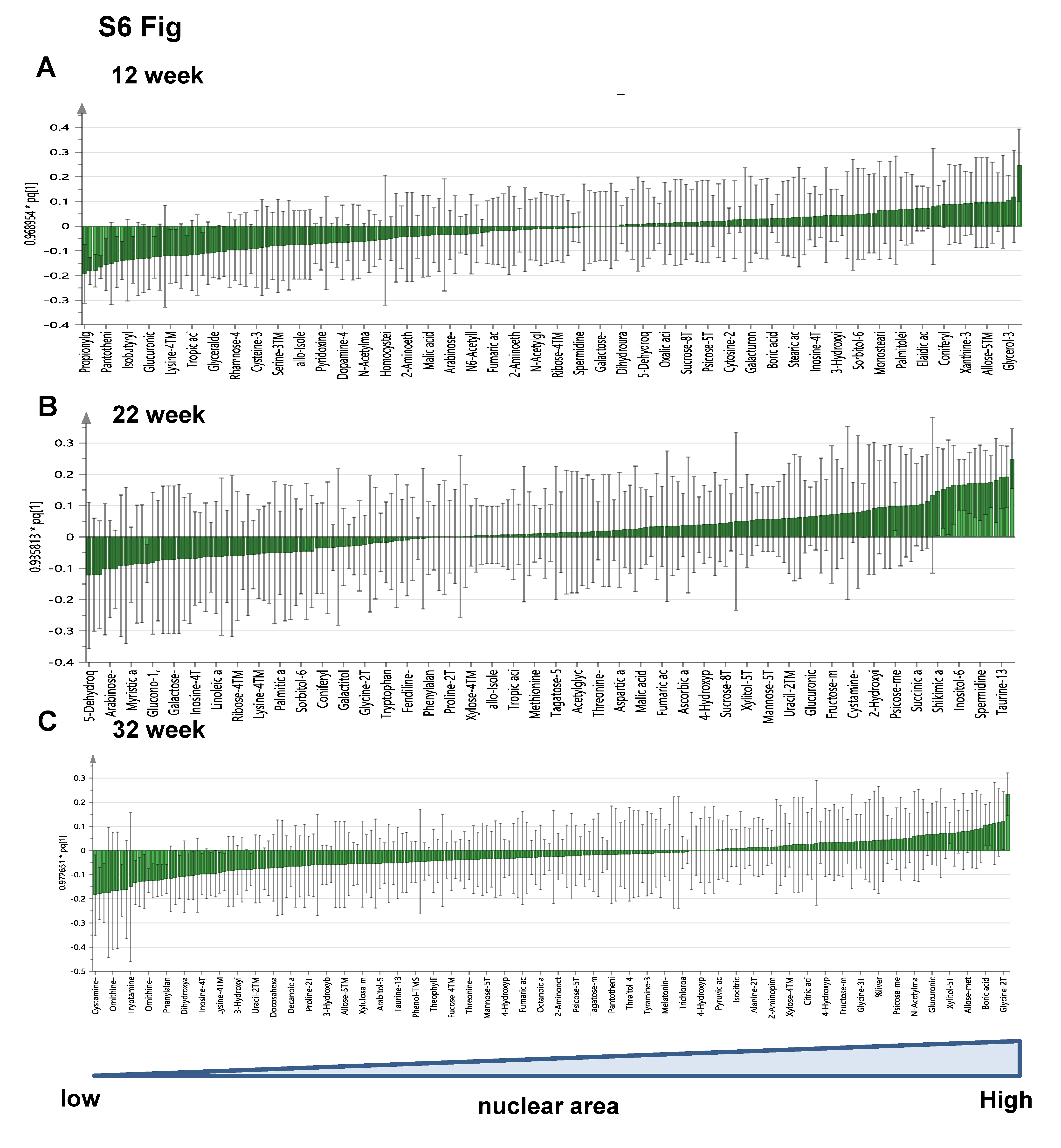

Supplement: S6 Fig — An OPLS analysis was performed to investigate metabolites with variable importance in projection for glomerular cellular component levels at 32 weeks in 12-week-old mice (A), 22-week-old mice (B), and 32-week-old mice (C). Loading column plots are shown. (TIF) [file pone.0219403.s006.tif]

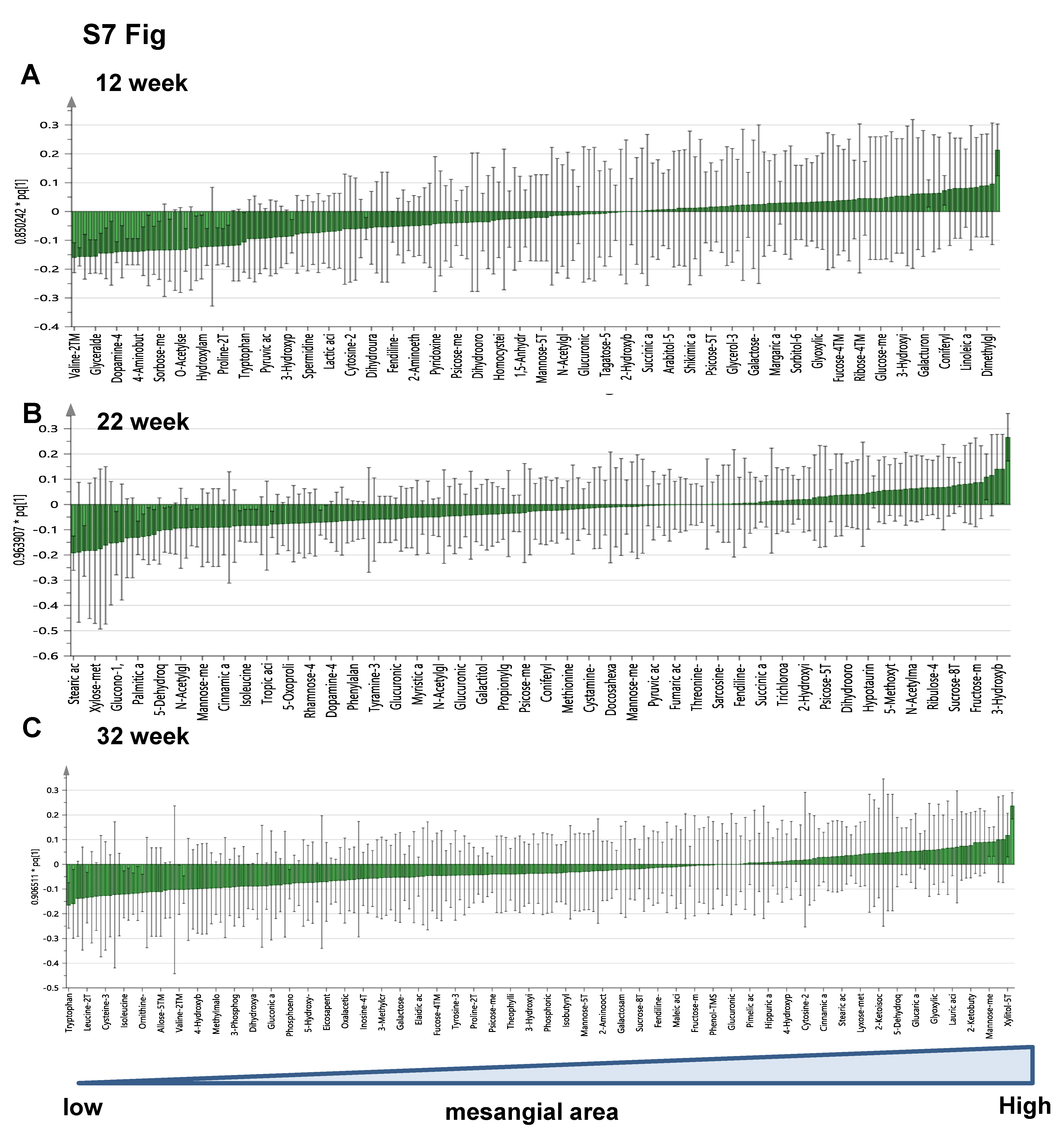

Supplement: S7 Fig — An OPLS analysis was performed to investigate metabolites with variable importance in projection for mesangial substrate levels at 32 weeks in 12-week-old mice (A), 22-week-old mice (B), and 32-week-old mice (C). Loading column plots are shown. (TIF) [file pone.0219403.s007.tif]
