# Supplementary material for: Use of gas chromatography mass spectrometry to elucidate metabolites predicting the phenotypes of IgA nephropathy in hyper IgA mice
Source: PLoS One. 2019 Jul 10;14(7):e0219403. doi: 10.1371/journal.pone.0219403 (PMC6619804; doi:10.1371/journal.pone.0219403)
Supplement: S1 Table — Metabolites that were significantly higher (A) or lower (B) in 12-week-old HIGA mice, compared with control Balb/c mice, are shown. The levels of metabolites are shown as the ratio to an internal standard (2-isopropylmalic acid at 25 μg/μL). (PDF) [file pone.0219403.s008.pdf]

S1 Table

A. Metabolites significantly higher in HIGA mice in 12 week old								
**Metabolites**	**Balb/c**			**HIGA mice**			**p-value**	**HIGA/Balb/c**
Glucuronic acid-5TMS(2)	0.00168	±	0.00009	0.00487	±	0.00030	<0.00001	2.89541
Mannose-meto-5TMS(1)	0.09830	±	0.00661	0.19699	±	0.01158	<0.00001	2.00393
Allose-meto-5TMS(1)	0.09652	±	0.00645	0.19148	±	0.01124	<0.00001	1.98378
Uracil-2TMS	0.00146	±	0.00014	0.00320	±	0.00016	<0.00001	2.19080
Xanthine-3TMS	0.00086	±	0.00050	0.00799	±	0.00124	0.00006	9.25704
Psicose-5TMS(1)	0.00080	±	0.00005	0.00156	±	0.00010	0.00007	1.94161
Inositol phosphate-7TMS	0.00606	±	0.00037	0.00930	±	0.00054	0.00010	1.53509
Gluconic acid-6TMS	0.00018	±	0.00003	0.00061	±	0.00008	0.00017	3.43576
2-Ketoisocaproic acid-meto-TMS(2)	0.00258	±	0.00009	0.00361	±	0.00014	0.00013	1.39916
Myristic acid-TMS	0.00653	±	0.00032	0.01191	±	0.00078	0.00015	1.82336
4-Aminobenzoic acid-2TMS	0.00007	±	0.00003	0.00121	±	0.00024	0.00038	18.03908
Xylitol-5TMS	0.00099	±	0.00005	0.00144	±	0.00008	0.00017	1.45141
Psicose-5TMS(2)	0.00060	±	0.00008	0.00135	±	0.00011	0.00039	2.23584
Alanine-2TMS	0.02142	±	0.00316	0.04543	±	0.00371	0.00051	2.12132
Glucuronic acid lactone-3TMS(1)	0.00156	±	0.00006	0.00245	±	0.00015	0.00061	1.57312
2-Aminoethanol-3TMS	0.04199	±	0.00215	0.05368	±	0.00171	0.00064	1.27865
Acetylglycine-TMS	0.04199	±	0.00215	0.05368	±	0.00171	0.00064	1.27865
Glucuronic acid-5TMS(1)	0.00118	±	0.00009	0.00198	±	0.00013	0.00073	1.68309
Tagatose-5TMS(1)	0.00079	±	0.00007	0.00144	±	0.00011	0.00103	1.81158
Glucose-5TMS(1)	2.06144	±	0.08080	2.40867	±	0.05217	0.00143	1.16844
N6-Acetyllysine-2TMS	0.01840	±	0.00128	0.02324	±	0.00067	0.00147	1.26333
Sorbitol-6TMS	0.00669	±	0.00076	0.01296	±	0.00115	0.00176	1.93684
Pantothenic acid-3TMS	0.00250	±	0.00016	0.00373	±	0.00022	0.00183	1.49035
Lyxose-4TMS(1)	0.00379	±	0.00045	0.01193	±	0.00221	0.00281	3.14900
Indol-3-acetic acid-TMS	0.02195	±	0.00138	0.02762	±	0.00090	0.00215	1.25844
Fucose-4TMS(1)	0.00113	±	0.00013	0.00199	±	0.00021	0.00226	1.76489
Inosine-4TMS	0.00043	±	0.00010	0.00867	±	0.00237	0.00415	20.18891
Galactitol-6TMS	0.00706	±	0.00071	0.01296	±	0.00115	0.00274	1.83617
Glyceric acid-3TMS	0.00245	±	0.00013	0.00343	±	0.00019	0.00308	1.40029
Coniferyl alcohol-2TMS	0.00299	±	0.00013	0.00403	±	0.00020	0.00320	1.34618
Glucuronic acid-meto-5TMS(1)	0.00517	±	0.00042	0.00793	±	0.00053	0.00323	1.53207
meso-Erythritol-4TMS	0.00070	±	0.00008	0.00121	±	0.00010	0.00360	1.72877
Lactic acid-13C3-2TMS	0.00000	±	0.00225	0.00000	±	0.00068	0.00593	N/A
Galactosamine-5TMS(1)	0.01327	±	0.00071	0.02215	±	0.00189	0.00442	1.66865
Urea-2TMS	8.30846	±	0.25516	10.26836	±	0.41073	0.00481	1.23589

Mannose-meto-5TMS(2)	4.43481	±	0.27487	5.37962	±	0.16488	0.00561	1.21304
Galactose-meto-5TMS(2)	4.43481	±	0.27487	5.37423	±	0.16474	0.00582	1.21183
Glucose-meto-5TMS(2)	4.43131	±	0.27460	5.36632	±	0.16465	0.00598	1.21100
Glycine-2TMS	0.00129	±	0.00017	0.00219	±	0.00019	0.00677	1.69908
N-Acetylglutamine-2TMS	0.09745	±	0.00599	0.11806	±	0.00402	0.00899	1.21146
Glucosamine-5TMS(1)	0.01298	±	0.00081	0.01579	±	0.00056	0.00979	1.21603
Linoleic acid-TMS	0.00675	±	0.00058	0.00967	±	0.00089	0.01265	1.43214
Lactic acid-2TMS	0.24797	±	0.03048	0.43608	±	0.04572	0.01316	1.75865
Ribulose-4TMS	0.00107	±	0.00015	0.00184	±	0.00019	0.01521	1.71585
Palmitoleic acid-TMS	0.00104	±	0.00021	0.00210	±	0.00035	0.01721	2.02758
Tyramine-3TMS	0.01086	±	0.00084	0.01306	±	0.00044	0.01817	1.20258
Lysine-4TMS	0.12678	±	0.00868	0.16878	±	0.01440	0.02193	1.33129
5-Methoxytryptamine-2TMS	0.00328	±	0.00063	0.01194	±	0.00347	0.02791	3.63407
Fructose-meto-5TMS(2)	0.00385	±	0.00028	0.00548	±	0.00044	0.02393	1.42280
5-Dehydroquinic acid-5TMS	0.00127	±	0.00015	0.00163	±	0.00007	0.02403	1.28379
Threonic acid-4TMS	0.00825	±	0.00039	0.01121	±	0.00088	0.03235	1.35953
1,5-Anhydro-glucitol-4TMS	0.01178	±	0.00033	0.01456	±	0.00083	0.03318	1.23604
2-Hydroxyisobutyric acid-2TMS	0.00111	±	0.00015	0.00194	±	0.00024	0.03354	1.73829
Dihydroorotic acid-3TMS	0.00274	±	0.00014	0.00368	±	0.00029	0.03904	1.34028
Galactose-5TMS(2)	0.20826	±	0.00699	0.25306	±	0.01377	0.03987	1.21511
Mannose-5TMS(2)	0.20826	±	0.00699	0.25306	±	0.01377	0.03987	1.21511
Allose-5TMS	0.20826	±	0.00699	0.25306	±	0.01377	0.03987	1.21511
Taurine-3TMS	0.16493	±	0.01459	0.27369	±	0.03420	0.04253	1.65940
Cystamine-nTMS	0.00074	±	0.00007	0.00102	±	0.00008	0.04399	1.36546
Taurine-13C2-3TMS	0.02870	±	0.00294	0.04830	±	0.00627	0.04648	1.68299
Cinnamic acid-TMS	0.00095	±	0.00008	0.00129	±	0.00011	0.04920	1.36381

B. Metabolites significantly lower in HIGA mice in 12 weeks old

Metabolites	Balb/c			HIGA mice			p-value	HIGA/Balb/c
Tyrosine-3TMS	0.00510	±	0.00035	0.00254	±	0.00019	<0.00001	0.49751
Coniferyl aldehyde-meto-TMS(1)	0.00217	±	0.00027	0.00104	±	0.00014	0.00059	0.48068
Tiglylglycine-TMS	0.00479	±	0.00049	0.00332	±	0.00030	0.01353	0.69166
Hypotaurine-3TMS	0.01620	±	0.00147	0.01207	±	0.00101	0.03020	0.74505
Oxalic acid-2TMS	0.00236	±	0.00053	0.00109	±	0.00013	0.05456	0.46263
3-Methylcrotonoylglycine -TMS	0.00455	±	0.00041	0.00331	±	0.00033	0.03748	0.72788
Tryptophan-3TMS	0.00118	±	0.00009	0.00068	±	0.00016	0.04356	0.57495

Data were mean ± SEM.
