# Supplementary material for: Use of gas chromatography mass spectrometry to elucidate metabolites predicting the phenotypes of IgA nephropathy in hyper IgA mice
Source: PLoS One. 2019 Jul 10;14(7):e0219403. doi: 10.1371/journal.pone.0219403 (PMC6619804; doi:10.1371/journal.pone.0219403)
Supplement: S2 Table — Metabolites that were significantly higher (A) or lower (B) in 22-week-old HIGA mice, compared with control Balb/c mice, are shown. The levels of metabolites are shown as the ratio to an internal standard (2-isopropylmalic acid at 25 μg/μL). (PDF) [file pone.0219403.s009.pdf]

S2 Table

A. Metabolites significantly higher in HIGA mice in 22 weeks old								
**Metabolites**	**Balb/c**			**HIGA mice**			**p-value**	**HIGA/Balb/c**
Glucuronic acid-5TMS(2)	0.00188	±	0.00013	0.00470	±	0.00024	<0.00001	2.49209
Xanthine-3TMS	0.00111	±	0.00043	0.00895	±	0.00098	<0.00001	8.03874
Inosine-4TMS	0.00029	±	0.00011	0.00546	±	0.00072	0.00001	18.56310
4-Aminobenzoic acid-2TMS	0.00015	±	0.00003	0.00136	±	0.00021	0.00005	8.90803
Threonic acid-4TMS	0.01194	±	0.00089	0.01850	±	0.00075	0.00004	1.54915
Sorbitol-6TMS	0.01114	±	0.00133	0.02119	±	0.00122	0.00007	1.90193
Xylitol-5TMS	0.00135	±	0.00007	0.00197	±	0.00008	0.00008	1.45456
Myristic acid-TMS	0.00773	±	0.00053	0.01285	±	0.00069	0.00010	1.66276
N-Acetylglutamine-2TMS	0.10443	±	0.00498	0.13913	±	0.00439	0.00011	1.33230
Galactitol-6TMS	0.01005	±	0.00173	0.02089	±	0.00132	0.00011	2.07812
Psicose-5TMS(1)	0.00103	±	0.00008	0.00169	±	0.00009	0.00012	1.64356
2-Aminoethanol-2TMS	0.00576	±	0.00080	0.01257	±	0.00096	0.00019	2.18168
Glucuronic acid-5TMS(1)	0.00132	±	0.00007	0.00249	±	0.00025	0.00041	1.88544
Glucose-5TMS(1)	2.17224	±	0.05205	2.63608	±	0.06862	0.00029	1.21353
Psicose-5TMS(3)	0.00120	±	0.00008	0.00166	±	0.00007	0.00085	1.37779
Acetylglycine-TMS	0.02843	±	0.00231	0.04503	±	0.00356	0.00095	1.58383
Inositol phosphate-7TMS	0.00550	±	0.00031	0.00962	±	0.00073	0.00099	1.75006
meso-Erythritol-4TMS	0.00098	±	0.00003	0.00138	±	0.00010	0.00141	1.41240
Glucuronic acid-meto-5TMS(1)	0.00711	±	0.00028	0.00957	±	0.00059	0.00151	1.34646
Tagatose-5TMS(1)	0.00104	±	0.00010	0.00154	±	0.00008	0.00175	1.47613
N-Acetylglutamine-3TMS	0.19915	±	0.00549	0.23193	±	0.00572	0.00178	1.16460
N6-Acetyllysine-2TMS	0.02183	±	0.00122	0.02708	±	0.00083	0.00190	1.24074
Galactose-meto-5TMS(2)	5.09365	±	0.28196	6.35333	±	0.20402	0.00195	1.24730
Glucose-meto-5TMS(2)	5.08942	±	0.28285	6.34223	±	0.20328	0.00201	1.24616
Mannose-meto-5TMS(2)	5.10482	±	0.28394	6.36115	±	0.20403	0.00203	1.24611
Galactose-5TMS(2)	0.21135	±	0.01054	0.26775	±	0.00979	0.00203	1.26687
Mannose-5TMS(2)	0.21135	±	0.01054	0.26775	±	0.00979	0.00203	1.26687
Allose-5TMS	0.21135	±	0.01054	0.26775	±	0.00979	0.00203	1.26687
Psicose-5TMS(2)	0.00100	±	0.00011	0.00154	±	0.00009	0.00218	1.54426
Glucosamine-5TMS(1)	0.01531	±	0.00104	0.01902	±	0.00054	0.00229	1.24245
Tyramine-3TMS	0.01290	±	0.00092	0.01653	±	0.00057	0.00231	1.28135
Galactosamine-5TMS(1)	0.01456	±	0.00085	0.02193	±	0.00142	0.00246	1.50628
Glucuronic acid lactone-3TMS(1)	0.00196	±	0.00012	0.00275	±	0.00021	0.00401	1.40717
Indol-3-acetic acid-TMS	0.02685	±	0.00148	0.03265	±	0.00106	0.00509	1.21621
Psicose-meto-5TMS(1)	0.00512	±	0.00027	0.00660	±	0.00030	0.00542	1.29007

Ascorbic acid-4TMS	0.00442	±	0.00020	0.00586	±	0.00042	0.00586	1.32793
Ribulose-4TMS	0.00114	±	0.00010	0.00179	±	0.00014	0.00561	1.57127
2-Aminoethanol-3TMS	0.02839	±	0.00233	0.04503	±	0.00356	0.00579	1.58605
Lyxose-4TMS(1)	0.00237	±	0.00041	0.00400	±	0.00031	0.00586	1.68796
Dihydroorotic acid-3TMS	0.00270	±	0.00017	0.00418	±	0.00045	0.00733	1.55121
2-Ketoisocaproic acid-meto-TMS(2)	0.00337	±	0.00025	0.00474	±	0.00029	0.00656	1.40926
Tropic acid-2TMS	0.00200	±	0.00008	0.00240	±	0.00008	0.00738	1.19815
Pyridoxine-3TMS	0.00554	±	0.00044	0.00677	±	0.00020	0.00834	1.22346
Taurine-13C2-3TMS	0.04192	±	0.00300	0.06270	±	0.00670	0.01146	1.49559
Fucose-4TMS(1)	0.00099	±	0.00011	0.00165	±	0.00015	0.01158	1.65778
N-Acetylglutamine-4TMS	0.02862	±	0.00143	0.03541	±	0.00156	0.01212	1.23701
1,5-Anhydro-glucitol-4TMS	0.01187	±	0.00063	0.01486	±	0.00069	0.01222	1.25176
Taurine-3TMS	0.23785	±	0.01658	0.34801	±	0.03620	0.01300	1.46317
Trichloroacetic acid-TMS	0.05384	±	0.00778	0.11005	±	0.01431	0.01570	2.04395
Lactic acid-2TMS	0.21056	±	0.03173	0.43481	±	0.05701	0.01572	2.06501
Mannose-meto-5TMS(1)	0.18147	±	0.02416	0.24739	±	0.01311	0.01657	1.36327
2-Hydroxyisobutyric acid-2TMS	0.00086	±	0.00018	0.00194	±	0.00028	0.01677	2.25805
Uracil-2TMS	0.00164	±	0.00015	0.00226	±	0.00015	0.01969	1.37760
Pantothenic acid-3TMS	0.00231	±	0.00027	0.00342	±	0.00028	0.02128	1.48009
Fructose-meto-5TMS(2)	0.00522	±	0.00032	0.00634	±	0.00027	0.02190	1.21456
5-Dehydroquinic acid-5TMS	0.00141	±	0.00010	0.00180	±	0.00010	0.02267	1.28189
Coniferyl alcohol-2TMS	0.00387	±	0.00017	0.00476	±	0.00024	0.02318	1.23151
Allose-meto-5TMS(1)	0.18026	±	0.02438	0.23626	±	0.01141	0.02733	1.31069
4-Hydroxyphenyllactic acid-3TMS	0.03306	±	0.00123	0.03720	±	0.00109	0.03092	1.12516
Succinic acid-2TMS	0.00370	±	0.00019	0.00588	±	0.00066	0.03391	1.59226
Pimelic acid-2TMS	0.10084	±	0.00131	0.10367	±	0.00066	0.04335	1.02810
Galactose-meto-5TMS(1)	4.73092	±	0.06733	5.62500	±	0.29592	0.04967	1.18899

B. Representative metabolites significantly lower in HIGA mice in 22 weeks old

Metabolites	Balb/c			HIGA mice			p-value	HIGA/Balb/c
Aspartic acid-3TMS	0.00281	±	0.00015	0.00207	±	0.00013	0.00279	0.73385
Coniferyl aldehyde-meto-TMS(1)	0.00228	±	0.00039	0.00121	±	0.00015	0.00568	0.53221
Tyrosine-3TMS	0.00595	±	0.00088	0.00327	±	0.00025	0.02270	0.54995
Threonine-3TMS	0.11375	±	0.01177	0.07815	±	0.00723	0.01401	0.68707
Glycine-3TMS	0.19522	±	0.00795	0.15976	±	0.00935	0.02415	0.81838
O-Acetylserine-2TMS	0.25075	±	0.02363	0.18547	±	0.01486	0.02483	0.73965

Isobutyrylglycine-TMS	0.01677	±	0.00146	0.01272	±	0.00093	0.02521	0.75886
Tryptophan-3TMS	0.00237	±	0.00042	0.00158	±	0.00016	0.04408	0.66721
Hypotaurine-3TMS	0.01736	±	0.00105	0.01372	±	0.00109	0.04874	0.79055

Data were mean ± SEM.
