# Supplementary material for: Use of gas chromatography mass spectrometry to elucidate metabolites predicting the phenotypes of IgA nephropathy in hyper IgA mice
Source: PLoS One. 2019 Jul 10;14(7):e0219403. doi: 10.1371/journal.pone.0219403 (PMC6619804; doi:10.1371/journal.pone.0219403)
Supplement: S3 Table — Metabolites that were significantly higher (A) or lower (B) in 32-week-old HIGA mice, compared with control Balb/c mice, are shown. The levels of metabolites are shown as the ratio to an internal standard (2-isopropylmalic acid at 25 μg/μL). (PDF) [file pone.0219403.s010.pdf]

S3 Table

A. Metabolites significantly higher in HIGA mice in 32 weeks old								
**Metabolites**	**Balb/c**			**HIGA mice**			**p-value**	**HIGA/Balb/c**
Glucuronic acid-5TMS(2)	0.00195	±	0.00013	0.00401	±	0.00011	<0.00001	2.06108
Leucine-2TMS	0.01156	±	0.00076	0.03006	±	0.00269	0.00001	2.59975
Alanine-2TMS	0.01485	±	0.00116	0.03163	±	0.00224	0.00007	2.13050
Valine-2TMS	0.08835	±	0.00379	0.14761	±	0.01118	0.00013	1.67076
Inositol phosphate-7TMS	0.00365	±	0.00035	0.00675	±	0.00040	0.00009	1.85005
Xylitol-5TMS	0.00106	±	0.00010	0.00195	±	0.00012	0.00010	1.82945
Propionylglycine-TMS	0.01312	±	0.00068	0.01920	±	0.00105	0.00011	1.46358
Phenylalanine-2TMS	0.07975	±	0.00089	0.10720	±	0.00579	0.00038	1.34424
allo-Isoleucine-2TMS	0.00631	±	0.00041	0.01317	±	0.00144	0.00036	2.08706
Myristic acid-TMS	0.04126	±	0.00315	0.06338	±	0.00303	0.00022	1.53618
Methylmalonic acid-2TMS	0.00095	±	0.00005	0.00169	±	0.00016	0.00041	1.78943
Serine-3TMS	0.00691	±	0.00026	0.00933	±	0.00050	0.00041	1.34969
N-Acetylglutamine-4TMS	0.03096	±	0.00128	0.04200	±	0.00170	0.00042	1.35685
Psicose-5TMS(3)	0.00143	±	0.00005	0.00201	±	0.00010	0.00073	1.40097
Galactose-5TMS(2)	0.23864	±	0.00783	0.30036	±	0.01004	0.00074	1.25862
Mannose-5TMS(2)	0.23864	±	0.00783	0.30036	±	0.01004	0.00074	1.25862
Allose-5TMS	0.23864	±	0.00783	0.30036	±	0.01004	0.00074	1.25862
4-Aminobutyric acid-2TMS	0.00518	±	0.00036	0.01206	±	0.00168	0.00131	2.32631
Ascorbic acid-4TMS	0.00589	±	0.00045	0.00905	±	0.00070	0.00122	1.53595
Ornithine-4TMS	0.07833	±	0.00409	0.12792	±	0.01245	0.00170	1.63322
Galactosamine-5TMS(1)	0.01697	±	0.00088	0.02491	±	0.00148	0.00190	1.46790
Aconitic acid-3TMS	0.00026	±	0.00008	0.00080	±	0.00013	0.00211	3.00312
Isoleucine-2TMS	0.00736	±	0.00063	0.01285	±	0.00144	0.00278	1.74522
Glyceraldehyde-meto-2TMS(2)	0.00117	±	0.00016	0.00244	±	0.00025	0.00276	2.08044
5-Dehydroquinic acid-5TMS	0.00086	±	0.00010	0.00124	±	0.00006	0.00295	1.44342
Ornithine-d7-4TMS	0.00612	±	0.00060	0.01074	±	0.00122	0.00329	1.75493
Sorbose-meto-5TMS(1)	0.00476	±	0.00030	0.00614	±	0.00027	0.00562	1.29063
Tagatose-meto-5TMS(1)	0.00179	±	0.00010	0.00234	±	0.00011	0.00612	1.30427
Inosine-4TMS	0.00870	±	0.00169	0.01892	±	0.00290	0.00683	2.17511
Proline-2TMS	0.00551	±	0.00048	0.00865	±	0.00095	0.00859	1.57011
1,5-Anhydro-glucitol-4TMS	0.01396	±	0.00057	0.01723	±	0.00076	0.01073	1.23455
Galacturonic acid-meto-5TMS(1)	0.00199	±	0.00022	0.00305	±	0.00025	0.01284	1.53589
4-Hydroxyproline-3TMS	0.00060	±	0.00005	0.00098	±	0.00009	0.01290	1.64626
Glucosamine-5TMS(1)	0.01071	±	0.00093	0.01343	±	0.00062	0.02218	1.25321
Rhamnose-4TMS(1)	0.00102	±	0.00009	0.00128	±	0.00006	0.03240	1.24794
Indol-3-acetic acid-TMS	0.01979	±	0.00142	0.02354	±	0.00093	0.03450	1.18954

Galactitol-6TMS	0.00917	± 0.00137	0.01325	± 0.00107	0.03552	1.44513
Coniferyl alcohol-2TMS	0.00354	± 0.00032	0.00422	± 0.00014	0.03570	1.19172
Tyramine-3TMS	0.00914	± 0.00074	0.01140	± 0.00061	0.03695	1.24799
2-Methylhippuric acid-TMS	0.00249	± 0.00051	0.00421	± 0.00051	0.04642	1.69078

B. Metabolites significantly lower in HIGA mice in 32 weeks old

Metabolites	Balb/c		HIGA mice		p-value	HIGA/Balb/c
Phosphoenolpyruvic acid-3TMS	0.00536	± 0.00041	0.00095	± 0.00019	<0.00001	0.17784
2-Hydroxybutyric acid-2TMS	0.00243	± 0.00021	0.00075	± 0.00012	<0.00001	0.30716
Hypotaurine-3TMS	0.02321	± 0.00217	0.00813	± 0.00048	0.00033	0.35015
Glycerol 3-phosphate-4TMS	0.00827	± 0.00062	0.00411	± 0.00020	0.00030	0.49678
N-Acetylmannosamine-meto-4TMS(2)	0.05308	± 0.00458	0.02843	± 0.00171	0.00001	0.53559
O-Phosphoethanolamine-4TMS	0.00261	± 0.00025	0.00101	± 0.00008	0.00041	0.38569
Dopamine-4TMS	0.01491	± 0.00125	0.00728	± 0.00068	0.00001	0.48847
Oxalic acid-2TMS	0.00302	± 0.00031	0.00110	± 0.00011	0.00053	0.36407
Eicosapentaenoic acid-TMS	0.18088	± 0.01689	0.07780	± 0.00585	0.00054	0.43011
Dihydroxyacetone phosphate-meto-3TMS(1)	0.00147	± 0.00015	0.00054	± 0.00007	0.00043	0.36526
Ribose-meto-4TMS	0.00452	± 0.00035	0.00248	± 0.00020	0.00003	0.54858
Ribulose-meto-4TMS	0.00205	± 0.00017	0.00119	± 0.00008	0.00004	0.58098
Succinic acid-2TMS	0.00791	± 0.00087	0.00321	± 0.00023	0.00131	0.40651
Inositol-6TMS(2)	0.13121	± 0.01151	0.06931	± 0.00430	0.00112	0.52827
Dihydroxyacetone phosphate-meto-3TMS(2)	0.00122	± 0.00016	0.00040	± 0.00005	0.00131	0.32713
Dopa-4TMS	0.00415	± 0.00035	0.00249	± 0.00016	0.00008	0.60043
Phenol-TMS	0.00352	± 0.00052	0.00080	± 0.00015	0.00158	0.22860
Decanoic acid-TMS	0.01619	± 0.00125	0.01032	± 0.00056	0.00009	0.63751
Erythrulose-meto-3TMS(1)	0.72323	± 0.02069	0.59462	± 0.01544	0.00010	0.82217
Hydroxylamine-3TMS	0.05055	± 0.00244	0.03440	± 0.00199	0.00010	0.68048
Malic acid-3TMS	0.02395	± 0.00292	0.01106	± 0.00120	0.00011	0.46198
Fructose-meto-5TMS(2)	0.22478	± 0.00986	0.17473	± 0.00549	0.00012	0.77734
Galactose-meto-5TMS(1)	7.43521	± 0.28735	5.84642	± 0.18552	0.00013	0.78632
4-Hydroxyphenylpyruvic acid-meto-2TMS	0.20030	± 0.00870	0.15467	± 0.00522	0.00013	0.77218
Phosphoric acid-3TMS	5.62450	± 0.16438	4.89803	± 0.07155	0.00014	0.87084
Xylulose-meto-4TMS	0.00209	± 0.00019	0.00130	± 0.00007	0.00019	0.62428
3,6-Epoxydodecanedioic acid-2TMS	0.00105	± 0.00018	0.00034	± 0.00006	0.00019	0.32197
Acetylglycine-2TMS	0.00289	± 0.00020	0.00195	± 0.00011	0.00020	0.67335
Fumaric acid-2TMS	0.02455	± 0.00092	0.01601	± 0.00130	0.00036	0.65196

3-Phosphoglyceric acid-4TMS	0.00555	± 0.00034	0.00386	± 0.00022	0.00036	0.69496
Galactosamine-5TMS(2)	0.00891	± 0.00126	0.00334	± 0.00029	0.00400	0.37502
Melatonin-2TMS	2.24701	± 0.26526	1.09799	± 0.13955	0.00044	0.48865
3-Aminopropanoic acid-3TMS	0.00113	± 0.00015	0.00048	± 0.00005	0.00362	0.42646
Galacturonic acid-5TMS(1)	0.01320	± 0.00196	0.00485	± 0.00036	0.00498	0.36720
5-Methoxytryptamine-2TMS	0.02712	± 0.00332	0.01201	± 0.00201	0.00059	0.44282
4-Hydroxyphenyllactic acid-3TMS	0.05778	± 0.00278	0.04507	± 0.00186	0.00103	0.78001
Uracil-2TMS	0.00263	± 0.00033	0.00147	± 0.00014	0.00115	0.56102
2-Ketoglutaric acid-meto-2TMS	0.01814	± 0.00280	0.00837	± 0.00121	0.00130	0.46158
Dihydrouracil-TMS	0.02307	± 0.00091	0.01922	± 0.00057	0.00140	0.83330
Glucose-meto-5TMS(1)	15.6395	± 0.57804	13.6673	± 0.26261	0.00190	0.87390
2-Ketobutyric acid-meto-TMS(1)	0.00287	± 0.00014	0.00174	± 0.00021	0.00202	0.60706
3-Hydroxyisovaleric acid-2TMS	0.00161	± 0.00012	0.00120	± 0.00006	0.00248	0.74937
Niacinamide-TMS	0.00512	± 0.00077	0.00247	± 0.00016	0.01307	0.48333
Hypoxanthine-2TMS	0.00953	± 0.00096	0.00483	± 0.00090	0.00432	0.50710
Aspartic acid-3TMS	0.00182	± 0.00017	0.00108	± 0.00014	0.00512	0.59206
Mannose 6-phosphate-meto-6TMS(1)	0.00105	± 0.00025	0.00025	± 0.00004	0.02029	0.24148
Glycerol-3TMS	0.20957	± 0.02171	0.13318	± 0.01499	0.00879	0.63548
Taurine-3TMS	0.60600	± 0.11676	0.26885	± 0.01358	0.02766	0.44365
Spermidine-5TMS	0.00186	± 0.00026	0.00071	± 0.00025	0.01039	0.38103
3-Methylcrotonoylglycine-TMS	0.00550	± 0.00095	0.00264	± 0.00032	0.02361	0.47955
Taurine-13C2-3TMS	0.10576	± 0.02040	0.04757	± 0.00239	0.02895	0.44976
Xanthine-3TMS	0.01033	± 0.00098	0.00703	± 0.00076	0.01886	0.68063
2-Hydroxyisobutyric acid-2TMS	0.00168	± 0.00025	0.00099	± 0.00015	0.02116	0.59212
Stearic acid-TMS	1.11677	± 0.05147	0.94987	± 0.04058	0.02408	0.85055
Lactic acid-2TMS	0.34295	± 0.04000	0.22479	± 0.02852	0.02690	0.65546
Trichloroacetic acid-TMS	0.08399	± 0.00923	0.05377	± 0.00777	0.02929	0.64022
Tyrosine-3TMS	0.00234	± 0.00009	0.00191	± 0.00016	0.03122	0.81751
4-Hydroxybutyric acid-2TMS	0.00924	± 0.00040	0.00784	± 0.00038	0.03263	0.84793
Glucose-5TMS(1)	3.61107	± 0.15596	3.27590	± 0.07543	0.04034	0.90718
Glyoxylic acid-meto-TMS	0.01206	± 0.00108	0.00842	± 0.00106	0.04410	0.69812
Monostearin-2TMS	0.06458	± 0.00315	0.05521	± 0.00266	0.04544	0.85486

Data were mean ± SEM.
