# Supplementary material for: Use of gas chromatography mass spectrometry to elucidate metabolites predicting the phenotypes of IgA nephropathy in hyper IgA mice
Source: PLoS One. 2019 Jul 10;14(7):e0219403. doi: 10.1371/journal.pone.0219403 (PMC6619804; doi:10.1371/journal.pone.0219403)
Supplement: S4 Table — Metabolites that were significantly higher (A) or lower (B) in both 12- and 22-week-old HIGA mice, compared with control Balb/c mice, are shown. (PDF) [file pone.0219403.s011.pdf]

S4 Table. Metabolites significantly modulated in HIGA mice in both 12 week and 22 week old.

**A. Metabolites significantly higher in HIGA mice in both 12 week and 22 week old.**				
	12 week old		22 week old	
**Metabolites**	**HIGA/Balb/c**	**p-value**	**HIGA/Balb/c**	**p-value**
Glucuronic acid-5TMS(2)	2.89541	<0.00001	2.49209	<0.00001
Mannose-meto-5TMS(1)	2.00393	<0.00001	1.36327	0.01657
Allose-meto-5TMS(1)	1.98378	<0.00001	1.31069	0.02733
Uracil-2TMS	2.19080	<0.00001	1.37760	0.01969
Xanthine-3TMS	9.25704	0.00006	8.03874	<0.00001
Psicose-5TMS(1)	1.94161	0.00007	1.64356	0.00012
Inositol phosphate-7TMS	1.53509	0.00010	1.75006	0.00099
2-Ketoisocaproic acid-meto-TMS(2)	1.39916	0.00013	1.40926	0.00656
Myristic acid-TMS	1.82336	0.00015	1.66276	0.00010
4-Aminobenzoic acid-2TMS	18.03908	0.00038	8.90803	0.00005
Xylitol-5TMS	1.45141	0.00017	1.45456	0.00008
Psicose-5TMS(2)	2.23584	0.00039	1.54426	0.00218
Glucuronic acid lactone-3TMS(1)	1.57312	0.00061	1.40717	0.00401
2-Aminoethanol-3TMS	1.27865	0.00064	1.58605	0.00579
Acetylglycine-TMS	1.27865	0.00064	1.58383	0.00095
Glucuronic acid-5TMS(1)	1.68309	0.00073	1.88544	0.00041
Tagatose-5TMS(1)	1.81158	0.00103	1.47613	0.00175
Glucose-5TMS(1)	1.16844	0.00143	1.21353	0.00029
N6-Acetyllysine-2TMS	1.26333	0.00147	1.24074	0.00190
Sorbitol-6TMS	1.93684	0.00176	1.90193	0.00007
Pantothenic acid-3TMS	1.49035	0.00183	1.48009	0.02128
Lyxose-4TMS(1)	3.14900	0.00281	1.68796	0.00586
Indol-3-acetic acid-TMS	1.25844	0.00215	1.21621	0.00509
Fucose-4TMS(1)	1.76489	0.00226	1.65778	0.01158
Inosine-4TMS	20.18891	0.00415	18.56310	0.00001
Galactitol-6TMS	1.83617	0.00274	2.07812	0.00011
Coniferyl alcohol-2TMS	1.34618	0.00320	1.23151	0.02318
Glucuronic acid-meto-5TMS(1)	1.53207	0.00323	1.34646	0.00151
meso-Erythritol-4TMS	1.72877	0.00360	1.41240	0.00141
Galactosamine-5TMS(1)	1.66865	0.00442	1.24245	0.00229
Mannose-meto-5TMS(2)	1.21304	0.00561	1.24611	0.00203
Galactose-meto-5TMS(2)	1.21183	0.00582	1.24730	0.00195
Glucose-meto-5TMS(2)	1.21100	0.00598	1.24616	0.00201
N-Acetylglutamine-2TMS	1.21146	0.00899	1.33230	0.00011

Glucosamine-5TMS(1)	1.21603	0.00979	1.50628	0.00246
Lactic acid-2TMS	1.75865	0.01316	2.06501	0.01572
Ribulose-4TMS	1.71585	0.01521	1.57127	0.00561
Tyramine-3TMS	1.20258	0.01817	1.28135	0.00231
Fructose-meto-5TMS(2)	1.42280	0.02393	1.21456	0.02190
5-Dehydroquinic acid-5TMS	1.28379	0.02403	1.28189	0.02267
Threonic acid-4TMS	1.35953	0.03235	1.54915	0.00004
1,5-Anhydro-glucitol-4TMS	1.23604	0.03318	1.25176	0.01222
2-Hydroxyisobutyric acid-2TMS	1.73829	0.03354	2.25805	0.01677
Dihydroorotic acid-3TMS	1.34028	0.03904	1.55121	0.00733
Galactose-5TMS(2)	1.21511	0.03987	1.26687	0.00203
Mannose-5TMS(2)	1.21511	0.03987	1.26687	0.00203
Allose-5TMS	1.21511	0.03987	1.26687	0.00203
Taurine-3TMS	1.65940	0.04253	1.46317	0.01300
Taurine-13C2-3TMS	1.68299	0.04648	1.49559	0.01146

B. Metabolites significantly lower in HIGA mice in both 12 week and 22 week old.

	12 week old		22 week old	
**Metabolites**	**HIGA/Balb/c**	**p-value**	**HIGA/Balb/c**	**p-value**
Tyrosine-3TMS	0.49751	<0.00001	0.54995	0.02270
Coniferyl aldehyde-meto-TMS(1)	0.48068	0.00059	0.53221	0.00568
Hypotaurine-3TMS	0.74505	0.03020	0.79055	0.04874
Tryptophan-3TMS	0.57495	0.04356	0.66721	0.04408
